# Test–retest reliability of approach‐avoidance conflict decision‐making during functional magnetic resonance imaging in healthy adults

**DOI:** 10.1002/hbm.25371

**Published:** 2021-03-02

**Authors:** Timothy J. McDermott, Namik Kirlic, Elisabeth Akeman, James Touthang, Ashley N. Clausen, Rayus Kuplicki, Robin L. Aupperle

**Affiliations:** ^1^ Laureate Institute for Brain Research Tulsa Oklahoma USA; ^2^ Department of Psychology University of Tulsa Tulsa Oklahoma USA; ^3^ Kansas City VA Medical Center Kansas City Missouri USA; ^4^ Department of Community Medicine University of Tulsa Tulsa Oklahoma USA

**Keywords:** affect, anxiety, fMRI, ICC, neural, psychiatry, translation

## Abstract

Neural and behavioral mechanisms during approach‐avoidance conflict decision‐making are relevant across various psychiatric disorders, particularly anxiety disorders. Studies using approach‐avoidance conflict paradigms in healthy adults have identified preliminary neural mechanisms, but findings must be replicated and demonstrated as reliable before further application. This study sought to replicate previous findings and examine test–retest reliability of behavioral (approach behavior, reaction time) and neural (regions of interest [ROIs]) responses during an approach‐avoidance conflict task conducted during functional magnetic resonance imaging (fMRI). Thirty healthy adults completed an approach‐avoidance conflict task during fMRI on two occasions (mean interval: 17 days; range: 11–32). Effects of task condition during three task phases (decision‐making, affective outcome and monetary reward) and intraclass correlation coefficients (ICCs) were calculated across time points. Results replicated that approach behavior was modulated by conflict during decision‐making. ROI activations were replicated such that dorsal anterior cingulate cortex (dACC) was modulated by conflict during decision‐making, and dACC, striatum, and anterior insula were modulated by valence during affective outcomes (*p*'s <.0083). Approach behavior during conflict demonstrated excellent reliability (ICCs ≥.77). Activation of dACC during conflict decision‐making and anterior insula during negative outcomes demonstrated fair reliability (ICCs = .51 and .54), and dACC and striatum activation demonstrated good reliability during negative outcomes (ICCs = .63 and .69). Two additional ROIs (amygdala, left dorsolateral prefrontal cortex) showed good reliability during negative outcomes (ICCs ≥.60). These results characterize several specific behavioral and neuroimaging responses that are replicable and sufficiently reliable during approach‐avoidance conflict decision‐making to support future utility.

## Graphical abstract

The following article examines the test‐retest reliability of behavioral and neural responses to an approach‐avoidance conflict task during functional magnetic resonance imaging. Specific responses with sufficient reliability were characterized across multiple levels of analysis.

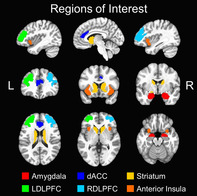

## 1 INTRODUCTION

Behavioral interactions with the environment are characterized by drives to approach situations with an opportunity for gaining reward and/or to avoid situations with a possibility for experiencing harm (Gray, 1981; Gray & McNaughton, 2000; Lang, Bradley, & Cuthbert, 1998). These drives often conflict under ambiguous circumstances, an experience referred to as *approach‐avoidance conflict* (Aupperle & Paulus, 2010; Quartz, 2009; Rolls & Grabenhorst, 2008). Approach‐avoidance conflict paradigms have been used extensively as animal models of anxiety (Kirlic, Young, & Aupperle, 2017; La‐Vu, Tobias, Schuette, & Adhikari, 2020), and these paradigms have been translated into human research to study the relevant neural and behavioral mechanisms (Aupperle, Melrose, Francisco, Paulus, & Stein, 2015; Aupperle, Sullivan, Melrose, Paulus, & Stein, 2011; Bach et al., 2014; Ironside et al., 2020; O'Neil et al., 2015; Wilborn, Kramer, Stevenson, & Dvorak, 2018; Zorowitz et al., 2019). Behavioral and neural responses during approach‐avoidance conflict are important factors contributing to psychiatric disorders with pronounced affective disturbance (Aupperle & Paulus, 2010). This is particularly true for anxiety and depressive disorders marked by dysregulated threat reactivity and/or reward responsivity (Britton, Lissek, Grillon, Norcross, & Pine, 2011; Van Meurs, Wiggert, Wicker, & Lissek, 2014). While studies using approach‐avoidance conflict paradigms have identified preliminary mechanisms, their findings must be replicated and demonstrated as reliable before further application.

There have been several tasks developed in recent years to assess human behavior and brain responses during approach‐avoidance conflict (Aupperle et al., 2011, 2015; Bach et al., 2014; Ironside et al., 2020; O'Neil et al., 2015; Wilborn et al., 2018; Zorowitz et al., 2019). Each task asks individuals to respond during situations with conflicting motivations to avoid negative affective outcomes and approach potential reward, but specific task behavior and contexts vary. Aupperle et al.' (2011, 2015) task has specifically been examined in relation to anxiety symptoms and traits, as well as during functional magnetic resonance imaging (fMRI). In a behavioral study of 95 healthy young adults who completed this task, the presence of conflict (situations with potential for both negative affective outcomes and reward) resulted in greater avoidance compared to trials with only the offer of reward outcomes and that, during conflict, greater levels of reward resulted in increases in approach behavior (Aupperle et al., 2011). In addition, women exhibited less approach behavior than men, and self‐reported reward seeking and anxiety sensitivity were both related to approach behavior on the task, though differently for men and women. In a study of 15 healthy adults who completed this same approach‐avoidance conflict paradigm during fMRI, activity in the following regions was significantly modulated during conflict trials compared to nonconflict trials: dorsal anterior cingulate cortex (dACC), right dorsolateral prefrontal cortex (dlPFC), right anterior insula, and bilateral striatum (Aupperle et al., 2015). Behavioral data collected concurrently with fMRI replicated findings from the previous behavioral study. In addition, negative affective stimulus outcomes following decision‐making led to increased neural activation in amygdala, bilateral striatum, bilateral dlPFC, and right anterior insula. No activation differences were found when comparing reward and nonreward processing in this task, perhaps due to immediate presentation of reward following affective outcomes. Prior work examining reward processing with different fMRI tasks has shown activation in regions such as striatum and dlPFC during reward processing (Wang, Smith, & Delgado, 2016). While these findings are in line with hypotheses concerning neural circuitry underlying the processing of reward, threat, and conflict, replication is needed in larger samples. In fact, to support optimal utility of human approach‐avoidance conflict paradigms (i.e., to assess individual differences in psychopathology and/or treatment effects), it is imperative to identify tasks that elicit replicable and reliable behavioral and neural responses at both the group and individual levels of analysis (Hajcak, Meyer, & Kotov, 2017; Infantolino, Luking, Sauder, Curtin, & Hajcak, 2018; Poldrack et al., 2016).

Test–retest reliability studies using fMRI seek to quantify the reliability of neural activity across multiple time points (e.g., different days; Bennett & Miller, 2010), and they often examine the agreement between these measurements by computing intraclass correlation coefficients (ICCs; Shrout & Fleiss, 1979). Guidelines (Fleiss, 1986) indicate that ICC interpretation goes as such: poor reliability (ICCs <.4), fair reliability (ICCs = .4–.59), good reliability (ICCs = .6–.74), and excellent reliability (ICCs ≥.75). While no test–retest reliability analyses of approach‐avoidance conflict decision‐making tasks have been reported thus far, a recent meta‐analysis examining the test–retest reliability of neural activation measured during fMRI tasks found an average ICC value of.397 across 90 studies (Elliott et al., 2020), indicating that task‐based fMRI measurements had overall poor reliability on average. Although this meta‐analysis concluded that task‐fMRI measures are not suitable for research examining biomarkers or individual differences neuroscience, potential solutions to this problem include improving fMRI task design (i.e., designing more reliable tasks; McDermott, Kirlic, & Aupperle, 2018) or identifying specific neural regions or patterns of activation that have at least fair reliability in existing fMRI tasks (Hassel et al., 2020; Lois, Kirsch, Sandner, Plichta, & Wessa, 2018; McDermott et al., 2020). Moreover, reliability analyses of behavioral measurements, including metrics such as accuracy and reaction time, have also demonstrated poor reliability for many tasks, particularly when contrasting between multiple task conditions (Enkavi et al., 2019). Thus, it is also necessary to examine test–retest reliability of behavior during fMRI task performance.

Test–retest reliability studies are complex regarding analysis and interpretation, particularly for fMRI paradigms. There may be expected practice or habituation effects for repeated measurement, and these effects should be accounted for using proper ICC estimates. Multiple different ICCs can be used for reliability analyses, but test–retest reliability is typically quantified using single‐measure ICCs (Weir, 2005; Yen & Lo, 2002). Single‐measure ICCs can be computed treating visit as a fixed (i.e., ICC[3, 1]) or random (i.e., ICC[2, 1]) effect. Both of these ICC computations have been frequently utilized in fMRI test–retest reliability studies (Bennett & Miller, 2010). However, as ICC(3, 1) accounts for fixed effects consistent across the group, it should be used if practice or habituation effects are expected. Additionally, since the ICC metric computes the ratio of between‐subjects variance over the sum of the between‐subjects and within‐subjects variance, this metric can be deflated by measurements with low between‐subjects variance, as is often the case for behavioral or neural data (Hedge, Powell, & Sumner, 2018; Infantolino et al., 2018).

The present study sought to both replicate prior findings and to examine the test–retest reliability of behavioral data and neural activation measured by fMRI during performance of an approach‐avoidance conflict task (Aupperle et al., 2015). Primary analyses of neural activation utilized a priori ROIs selected from an anatomical atlas (Fan et al., 2016), while supplementary analyses of neural activation utilized a whole‐brain approach. Our hypotheses for replication analyses in this study were that: (1) approach behavior would be modulated by task condition, that is, reduced approach behavior during conflict and greater approach behavior with increasing levels of potential reward, (2) dACC, right dlPFC, anterior insula, and striatum ROIs would show increased activation during conflict processing compared to nonconflict processing, (3) amygdala, bilateral dlPFC, anterior insula, and striatum ROIs would show increased activation during negative stimulus outcomes compared to positive stimulus outcomes, and (4) striatum and bilateral dlPFC ROIs would show increased activation during reward compared no reward processing. As this is the first study to examine the test–retest reliability of this task, hypotheses were based on prior test–retest reliability analyses of similar conflict or affect‐focused tasks performed during fMRI (Hassel et al., 2020; Lois et al., 2018; McDermott et al., 2020). Our hypotheses for test–retest reliability analyses were that: (1) behavioral measures would show at least fair reliability, and (2) neuroimaging analyses would identify a subset of ROIs with fair or good reliability.

## 2 METHODS

### 2.1 Subject selection

Forty healthy adults with no psychiatric diagnoses were recruited to participate in the present study (25 females; mean age = 29.40 years, range: 19–53). Participants completed the same fMRI protocol on two different dates approximately 2–3 weeks apart at time 1 (T1) and time 2 (T2). Participants were assessed for psychiatric diagnoses using the Mini‐International Neuropsychiatric Interview (M.I.N.I.; Sheehan et al., 1998) Version 7.0.2 for *DSM*‐5 (American Psychiatric Association, 2013). Exclusionary criteria included diagnosis of a psychiatric disorder, concurrent use of psychotropic medications, medical illness that would affect central nervous system function (e.g., neurological disease), history of significant head trauma, current substance abuse, and ferromagnetic implants. A total of 10 participants who were enrolled in the study were excluded: five of whom were excluded for having greater than 20% of their trials removed due to excessive motion at T1 or T2 (i.e., using a threshold of 0.3 mm for the average Euclidean Norm [ENORM] of motion parameters), three due to scanner acquisition errors at either T1 or T2, and one for not performing the task at T2 (i.e., no response on 76% of trials). Last, one participant's individual percent signal change (PSC) values for several ROIs and contrasts were consistently found to be outliers (3–4 standard deviations outside of the group mean) and were thus excluded from analyses.

The final sample included 30 healthy adult participants (19 females; mean age = 28.90 years, *SD* = 8.14, range: 19–51). Self‐reported racial and ethnic demographics of the final sample were as such: 20 participants identified as Non‐Hispanic White, three identified as Hispanic White or Latino, two identified as Black or African American, one identified as Asian, and four identified as Native American or American Indian. Mean level of education was 14.93 years (*SD* = 2.27). Participants provided informed consent and received monetary compensation for study procedures following the guidelines of the Western Institutional Review Board, who approved the study protocol. Research was conducted in accordance with the World Medical Association Declaration of Helsinki.

### 2.2 Experimental paradigm and stimuli

**FIGURE 1:**
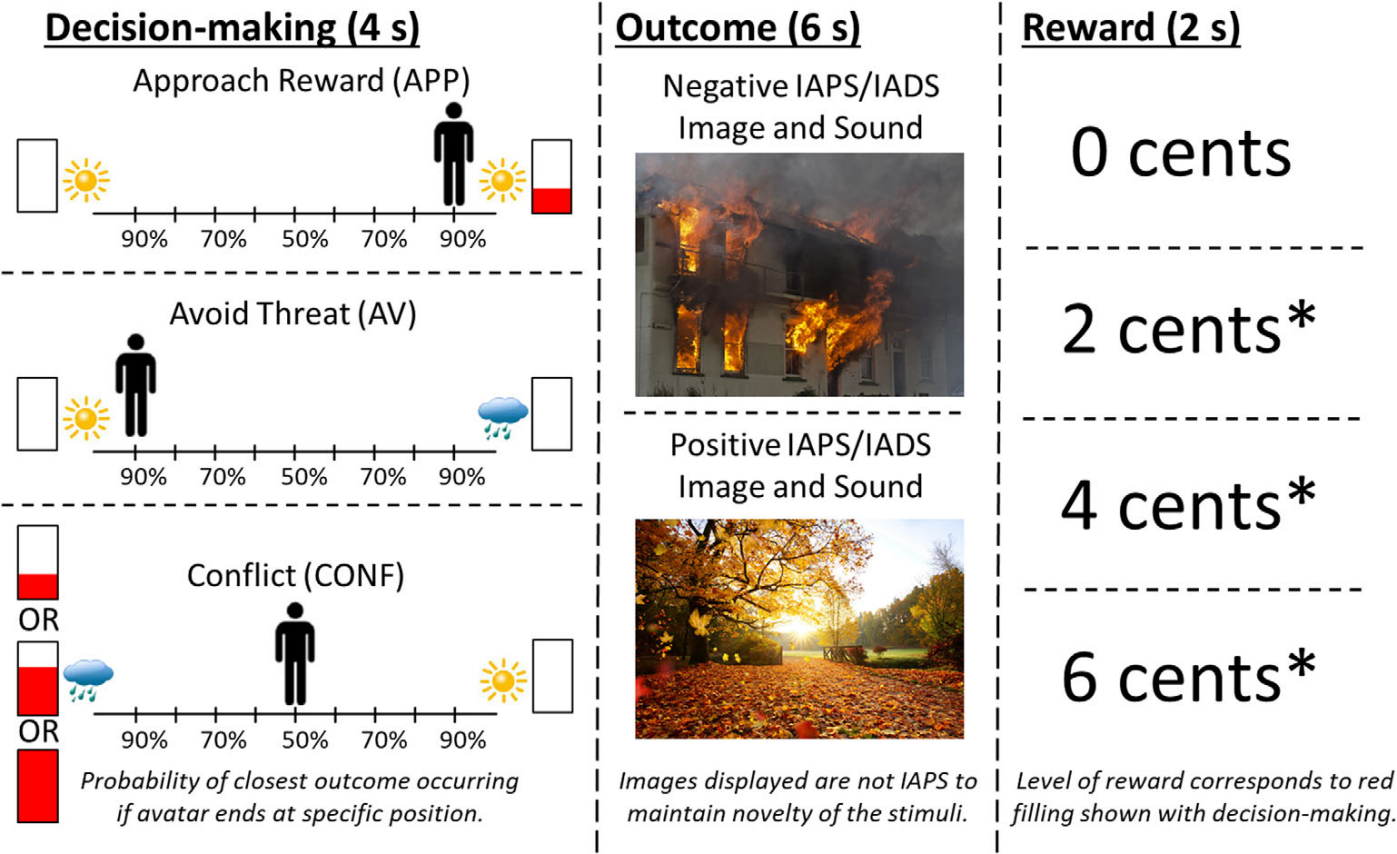
Approach‐avoidance conflict task. The three phases of the approach‐avoidance conflict task are displayed in order from left to right. (Left) During the decision‐making phase, participants have 4 s to move the avatar (by moving a joystick) to a position that accurately reflects their preference between the two potential outcomes. The position in which they move the avatar determines the relative probability of each of the two outcomes occurring (e.g., 90/10 or 50/50%). For approach reward (APP) trials, participants are presented with a choice of two positive stimuli outcomes, and one is paired with a 2‐cent reward as indicated by the filling of the red bar. For avoid threat trials (AV), participants are presented with a choice of a positive and negative stimulus outcome, and neither are paired with a reward. For conflict trials (CONF), participants are presented with a choice of a positive stimulus outcome not paired with a reward and a negative stimuli outcome that is paired with a reward. Reward level is indicated by the level of filling of the red bar, and this indicates either a 2‐, 4‐, or 6‐cent reward. (Middle) During the affective outcome phase, participants are presented with either a positive or a negative affective stimuli image/sound pairing. The images and sounds presented are drawn from the International Affective Picture System (IAPS; Lang et al., 2008) and the International Affective Sound (IADS; Bradley & Lang, 1999), and other public domain audio files. Note that images displayed are not from IAPS in order to maintain stimuli novelty. (Right) During the monetary reward phase, participants are presented with text indicating level of reward for this trial (i.e., 0, 2, 4, or 6 cents), the total award accumulated thus far, and a trumpet sound when receiving a reward (indicated by “*”)

The approach‐avoidance conflict task was conducted as previously described (Aupperle et al., 2015) and is detailed in Supporting Information. See Figure 1 for visualization. Briefly, the task consisted of three phases: (1) decision‐making, (2) affective outcome, and (3) monetary reward. During decision‐making, participants were presented with a runway that had pictures on each side to represent two possible outcomes (Figure 1). Possible outcomes included both an image (i.e., sun or cloud) indicative of an affective stimulus and a level of monetary reward (i.e., 0, 2, 4, or 6 in United States' cents) on each side. The image of a sun indicated a positively valenced stimulus outcome, and a cloud indicated a negatively valenced stimulus outcome. Level of reward was indicated by the amount of red ink filling a rectangular meter adjacent to the sun or cloud. For “approach‐reward” (APP) trials, there was no threat (i.e., no possibility of a negatively valenced outcome) and 2 cents offered on one side and positive affective outcomes on both sides. For “avoid‐threat” (AV) trials, there was no possibility of reward, with 0 cents offered for both a positive and negative affective outcome on each side. For “conflict” (CONF) trials, either 2 (CONF2), 4 (CONF4), or 6 (CONF6) cents were offered for negative affective outcomes while 0 cents were offered for positive affective outcomes. CONF trials induced approach‐avoidance conflict while APP and AV trials isolated approach/avoidance motivations. Participants used a joystick to move an avatar on the runway to indicate their preference for the potential affective outcomes, with the ending location determining the probability of each of the two affective outcomes (ranging from 10/90 to 90/10%, with the middle representing 50/50% chance of each). Avatar starting position was counterbalanced across trials. During the outcome phase, participants were presented with either positively or negatively valenced pictures and sounds that were drawn from the International Affective Picture System (IAPS; Lang, Bradley, & Cuthbert, 2008), the International Affective Digitized Sounds (IADS; Bradley & Lang, 1999), and other public domain audio files. During the reward phase, participants were given 0, 2, 4, or 6 cents (unlike previous work with this same task that used points [Aupperle et al., 2011, 2015]), and different tones played depending whether a reward was given or not.

The task used an event‐related design with 90 trials total (18 of each trial type: AV, APP, CONF2, CONF4, and CONF6) over three fMRI scans (i.e., 30 trials, or 480 s per scan). The stimulus presentation software used was PsychoPy (Version 1.84.2). Prior to entering the scanner and performing the task, participants received detailed instructions and completed four practice trials to ensure sufficient understanding. Practice stimuli were included in the sample of stimuli during the main task. The full sample of affective images and sounds was the same across the fMRI scans at each time point. However, the individual set of affective stimuli differed for each of the three fMRI runs, and the block order was randomized for each time point. Additionally, the specific outcomes that individuals were exposed to differed based on the choices they made during the decision‐making phase. The decision‐making phase lasted 4 s, affective outcome phase lasted 6 s, reward presentation phase lasted 2 s, and the intertrial interval lasted from 1 to 7 s(mean = 4 s). Task performance was measured through (1) *approach behavior* and (2) *reaction time*. Approach behavior was measured by the avatar's end position on the runway in relation to the negative outcome and/or reward, and this ranged from −4 (full avoidance from the negative outcome and/or reward) to +4 (full approach to the negative outcome and/or reward). Reaction time (RT) was defined as when participants initially moved the joystick during the decision‐making phase (i.e., first avatar position change). Approach behavior and RT were calculated for each participant and averaged by trial type. Due to a software error in the joystick configuration, RT data were unavailable for three subjects. These subjects approach behavior and imaging data were still usable, and thus, they were still included in analyses of all non‐RT data.

### 2.3 FMRI data acquisition and imaging parameters

Functional and structural images were acquired using a Discovery MR750 whole‐body 3.0 Tesla MRI scanner (GE Healthcare, Milwaukee, WI). A receive‐only 8‐element phased array coil (GE Healthcare) optimized for parallel imaging was used for MRI signal reception. During task performance, three fMRI scans collected BOLD signal using single‐shot, gradient‐recalled echo‐planar imaging (EPI) sequences with sensitivity encoding (96 × 96 matrix, 240 mm field of view [FOV], 1.875 × 1.875 mm^2^ in‐plane resolution, 39 axial slices, 2.9 mm slice thickness, 2.0 s repetition time [TR], 27 ms echo time [TE], 40° flip angle, 250 kHz sampling bandwidth, 256 volumes, and SENSE acceleration factor R = 2 in the phase‐encoding direction). One T1‐weighted Magnetization Prepared Rapid Gradient Echo (MPRAGE) imaging sequence with SENSE was used for anatomical reference and alignment purposes (256 × 256 matrix size, 240 mm FOV, 0.938 × 0.938 mm^2^ in‐plane resolution, 1.0 mm slice thickness, 5.94 ms TR, 1.96 ms TE, 8° flip angle, 31.2 kHz sampling bandwidth, 186 axial slices per volume, and acceleration factor R = 2).

### 2.4 Data preprocessing and subject‐level analyses

All structural and functional imaging data were preprocessed and analyzed using the Analysis of Functional NeuroImages (AFNI) software package (Cox, 1996). The first three volumes were discarded, and slice timing correction was performed for each volume. The anatomical image was aligned to an EPI image and warped to the MNI152_T1_2009c T1‐weighted anatomical template. EPI images were realigned to the first volume, normalized to the template image, and resampled to a voxel size of 2 × 2 × 2 mm^3^. Anatomical data were resampled to a voxel size of 1 × 1 × 1 mm^3^.

Individual participant time series data were analyzed using AFNI's 3dDeconvolve program (using a gamma variate hemodynamic response function [i.e., AFNI's “BLOCK” function]) with nine regressors of interest: AV, APP, CONF2, CONF4, and CONF6 decision‐making blocks, negative and positive affective stimuli outcome blocks, and reward and no reward blocks. Regressors of noninterest included motion parameters (*x*, *y*, and *z* translations; roll, pitch, and yaw rotations); baseline, linear, and quadratic trends; and the average time series from a mask of each individual's ventricles [constructed using the FreeSurfer Software Suite (Fischl, 2012)]. Regression coefficients were divided by the baseline regressor to calculate PSC. Last, a Gaussian filter with 4 mm full‐width at half maximum was applied. In our previous fMRI study using this task (Aupperle et al., 2015), PSC was combined across CONF2, CONF4, and CONF6 decision‐making trials for a single conflict condition (i.e., CONF), and PSC was also combined across APP and AV decision‐making trials for a single nonconflict condition (i.e., NONCONF). These combined CONF and NONCONF contrasts were then compared to model the effect of conflict. In the present study, ROI analyses examining condition effects during the decision‐making phase utilized an approach that separated the decision‐making conditions (APP, AV, CONF2, CONF4, and CONF6), while whole‐brain analyses utilized the combined approach to simplify interpretation (CONF vs. NONCONF).

### 2.5 ROI selection

**FIGURE 2:**
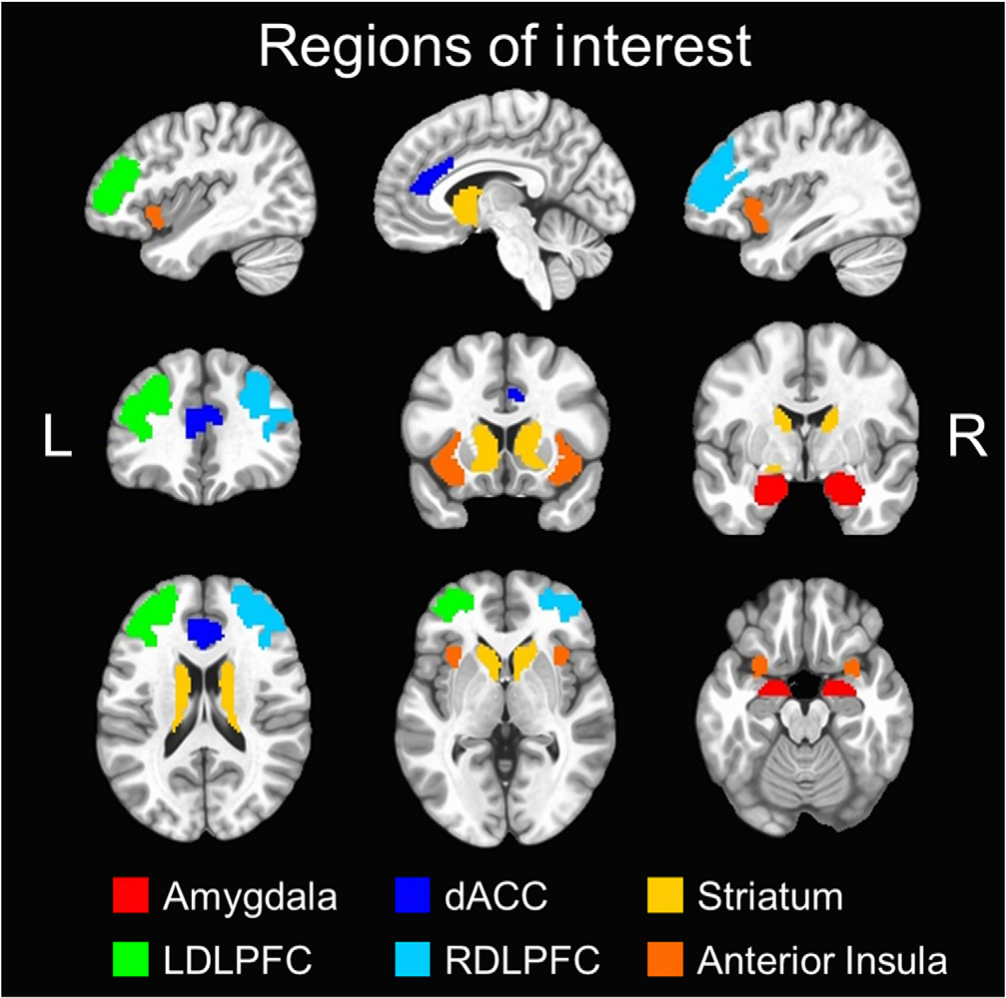
Brainnetome composite regions of interest. six composite regions of interest (ROIs) were used for primary analyses and were constructed using the Brainnetome atlas (Fan et al., 2016). These are overlaid on the MNI152_T1_2009c T1‐weighted anatomical template brain in neurological orientation (i.e., left is left) using the following color scheme: amygdala (red), dorsal anterior cingulate cortex (dark blue), striatum (orange), left dorsolateral prefrontal cortex (LDLPFC; green), right dorsolateral prefrontal cortex (RDLPFC; light blue), and anterior insula (yellow). The color legend for each ROI is above

A priori composite ROIs were constructed using subregions of the Brainnetome atlas (Fan et al., 2016; atlas.brainnetome.org). The Brainnetome atlas is an open‐access resource that provides a map of anatomical subregions of the human brain. These subregions were constructed using a comprehensive, multimodal neuroimaging approach that utilized both structural and functional connectivity information in addition to standard structural imaging (Fan et al., 2016). The Brainnetome atlas was utilized in the present study due to its basis in both structural and functional neuroimaging and the availability of subregion specificity within cortical and subcortical regions. These composite ROIs overlapped with clusters identified in the previous fMRI study using the approach‐avoidance conflict task (Aupperle et al., 2015). A total of six ROIs were constructed (see Figure 2), including bilateral amygdala (4 subregions), bilateral dACC (4 subregions), bilateral striatum (6 subregions), left dlPFC (3 subregions), right dlPFC (3 subregions), and bilateral anterior insula (4 subregions). Left and right dlPFC ROIs were separated to account for laterality effects observed in the Aupperle et al. (2015) study. Primary analyses used mean PSC data extracted from all voxels within each of the six composite ROIs. Mean PSC data were also extracted from voxels within individual ROI subregions for supplementary analyses.

### 2.6 Behavioral and ROI statistical analyses

Statistical analyses were carried out using the R Statistical Package (R Core Team, 2013), and ICC calculations were conducted using the R package “irr” (v0.84.1; Gamer, Lemon, Fellows, & Singh, 2019). Behavioral and ROI findings during the decision‐making phase of this task were examined using 5 × 2 within‐subjects ANOVAs to probe the effects of condition (APP, AV, CONF2, CONF4, and CONF6) and time (T1 and T2) as categorical factors. For ROI findings during the outcome and reward phases of this task, these were examined using 2 × 2 within‐subjects ANOVAs to probe the effects of condition (i.e., negative vs. positive image/sound outcomes; reward vs. no‐reward) and time (T1 and T2). To account for multiple comparisons across the six composite ROIs, a Bonferroni‐corrected *α* threshold of *p* <.0083 was used for within‐subjects ANOVAs of ROI data. Within‐subjects ANOVAs that were significant at *p* <.05 are still reported, but these are denoted as not meeting the adjusted threshold due to multiple comparisons correction. Follow‐up pairwise comparisons were still considered significant at *p* <.05.

Test–retest reliability of behavioral and ROI data across T1 and T2 was estimated using ICC(3, 1) for each measure. ICC(3, 1) was utilized in order to account for potential practice or habituation effects. To be comprehensive, test–retest reliability estimates of RT and ROI data during decision‐making were calculated for each of the five trial types separately, combined across the three CONF trial types (i.e., CONF2, CONF4, CONF6), combined across the two NONCONF trial types (i.e., AP, AV), and combined across all five trial types. Test–retest reliability estimates for approach behavior during decision‐making were not calculated for separate APP or AV trials, across NONCONF trials, or across all five trial types. This was due to the expected lack of variability in approach behavior on NONCONF trials, which would have confounded the ICC estimates (Hedge et al., 2018). When reporting ICC point estimates, we denote whether ROIs met cutoffs for fair (.4–.6), good (.6–.75), or excellent (>.75) reliability based on the guidelines from Fleiss (1986). Additionally, 95% confidence intervals are reported with ICC point estimates in Tables 1 and 2. These intervals provide an indication of how precise these ICC estimates are likely to be. Supplementary analyses of test–retest reliability were also conducted by estimating ICC(3, 1) for individual mean PSC values across T1 and T2 separately for all composite ROI subregions, and these results are reported in Supporting Information.

**TABLE 1:** ICCs for behavioral measures and composite ROIs during decision‐making phase

	Decision‐making trial type							
Behavioral measures	Approach	Avoid	Conflict‐2	Conflict‐4	Conflict‐6	Conflict‐averaged	Nonconflict‐averaged	All‐averaged
Approach behavior	—	—	0.80** (*0.62–0.90*)	0.77** (*0.57–0.88*)	0.79** (*0.61–0.90*)	0.79** (*0.60–0.89*)	—	—
Reaction time^@^	0.49^#^ (*0.14–0.73*)	0.53^#^ (*0.20–0.76*)	0.48^#^ (*0.13–0.72*)	0.56^#^ (*0.23–0.77*)	0.44^#^ (*0.08–0.70*)	0.53^#^ (*0.20–0.76*)	0.56^#^ (*0.23–0.77*)	0.54^#^ (*0.20–0.76*)
*Composite ROI*								
Amygdala	−0.06 (*−0.40–0.31*)	−0.11 (*−0.45–0.26*)	−0.18 (*−0.50–0.19*)	0.11 (*−0.26–0.44*)	−0.01 (*−0.36–0.35*)	−0.15 (*−0.48–0.21*)	−0.04 (*−0.39–0.32*)	−0.13 (*−0.46–0.24*)
dACC	0.48^#^ (*0.15–0.71*)	0.43^#^ (*0.09–0.68*)	0.43^#^ (*0.09–0.68*)	0.34 (*−0.02–0.62*)	0.47^#^ (*0.13–0.70*)	0.51^#^ (*0.19–0.74*)	0.53^#^ (*0.22–0.75*)	0.61* (*0.32–0.79*)
Striatum	0.28 (*−0.08–0.58*)	0.38 (*0.03–0.65*)	0.27 (*−0.09–0.57*)	0.17 (*−0.20–0.49*)	0.30 (*−0.07–0.59*)	0.47^#^ (*0.14–0.71*)	0.38 (*0.03–0.65*)	0.59^#^ (*0.30–0.78*)
Left dlPFC	0.36 (*0.00–0.63*)	0.65* (*0.38–0.81*)	0.38 (*0.03–0.65*)	0.37 (*0.02–0.64*)	0.26 (*−0.10–0.57*)	0.48^#^ (*0.15–0.72*)	0.54^#^ (*0.22–0.75*)	0.47^#^ (*0.14–0.71*)
Right dlPFC	0.38 (*0.03–0.65*)	0.55^#^ (*0.25–0.76*)	0.44^#^ (*0.11–0.69*)	−0.03 (*−0.38–0.33*)	0.11 (*−0.26–0.45*)	0.28 (*−0.08–0.58*)	0.51^#^ (*0.19–0.73*)	0.39 (*0.04–0.66*)
Anterior insula	0.06 (*−0.30–0.41*)	0.19 (*−0.18–0.51*)	0.13 (*−0.24–0.46*)	0.15 (*−0.22–0.48*)	−0.04 (*−0.39–0.32*)	0.11 (*−0.25–0.45*)	0.08 (*−0.28–0.43*)	0.14 (*−0.22–0.47*)

*Note*: ICCs are consistent agreement and single‐measure (i.e., ICC[3,1]). ICCs between .4 and .6 are denoted with “^#^.” ICCs between .6 and .75 are denoted with “*.” ICCs >.75 are denoted with “**.” Initial reaction time ICC estimates included data from 27 out of the 30 participants and thus are denoted with a ^@^. ICC value interpretation: poor (<.40), fair (.40–.59), good (.60–.74), and excellent (≥.75). 95% confidence intervals are provided in parentheses below each ICC estimate and are italicized. Negative ICCs are interpreted as having zero reliability.

Abbreviations: dACC, dorsal anterior cingulate cortex; dlPFC, dorsolateral prefrontal cortex; ICC, intraclass correlation coefficient; ROI, region of interest.

**TABLE 2:** ICCs for composite ROIs during outcome and reward phases

	Outcome phase		Reward phase	
Composite ROI	Negative	Positive	Reward	No reward
Amygdala	0.60* (*0.31–0.79*)	0.59^#^ (*0.29–0.78*)	−0.03 (*−0.38–0.33*)	0.21 (*−0.15–0.53*)
dACC	0.63* (*0.35–0.80*)	0.26 (*−0.11–0.56*)	0.41^#^ (*0.06–0.67*)	0.13 (*−0.24–0.46*)
Striatum	0.69* (*0.45–0.84*)	0.60* (*0.30–0.78*)	0.58^#^ (0.28–0.77)	0.19 (*−0.18–0.51*)
Left dlPFC	0.66* (*0.40–0.82*)	0.32 (*−0.04–0.61*)	0.14 (*−0.23–0.47*)	0.16 (*−0.21–0.49*)
Right dlPFC	0.49^#^ (*0.16–0.72*)	0.27 (*−0.09–0.57*)	0.27 (*−0.10–0.57*)	0.21 (*−0.16–0.53*)
Anterior insula	0.54^#^ (*0.23–0.75*)	0.15 (*−0.22–0.48*)	0.31 (*−0.05–0.60*)	0.20 (*−0.17–0.52*)

*Note*: ICCs are consistent agreement and single‐measure (i.e., ICC[3,1]). ICCs between .4 and .6 are denoted with “^#^”. ICCs between .6 and.75 are denoted with “*.” ICC value interpretation: poor (<.40), fair (.40–.59), good (.60–.74), excellent (≥.75). 95% confidence intervals are provided in parentheses below each ICC estimate and are italicized Negative ICCs are interpreted as having zero reliability.

Abbreviations: dACC, dorsal anterior cingulate cortex; dlPFC, dorsolateral prefrontal cortex; ICC, intraclass correlation coefficient; ROI, region of interest.

We also conducted additional supplementary analyses of ROI data to examine the following questions about reliability: (1) does the test–retest interval (i.e., days between scans) relate to test–retest reliability? and (2) does higher level of absolute mean PSC at T1 relate to greater test–retest reliability estimates? Detailed methods and results for these additional analyses are provided in Supporting Information.

### 2.7 Whole‐brain voxel‐wise statistical analyses

In addition to the ROI‐based approach, whole‐brain voxel‐wise analyses were also conducted. Whole‐brain within‐subjects ANOVAs were conducted using AFNI's 3dANOVA3 package to examine the effects of condition, time, and the condition‐by‐time interaction for each phase of the task. This led to a total of three ANOVAs, which compared CONF to NONCONF trial types (i.e., decision making phase), negative–positive outcomes (i.e., outcome phase), and reward‐no reward trials (i.e., reward phase). Results were statistically thresholded at *p* <.01, corrected for multiple comparisons using cluster‐based permutation testing with AFNI's 3dClustsim package (*α* <.05, resulting in voxel thresholds of 468, 591, and 498 for decision‐making, outcome, and reward phases, respectively). Last, to examine test–retest reliability, whole‐brain voxel‐wise ICCs were estimated using AFNI's 3dICC package separately for each of the task contrasts during decision‐making (i.e., CONF, NONCONF), outcome (i.e., negative, positive), and reward (i.e., reward, no reward) phases of the task. ICC(3,1) was calculated, with time as a fixed‐factor and subject as a random factor. These ICC maps were thresholded at a threshold of 0.4 to display voxels that had at least fair reliability. In addition to whole‐brain results reported, the outputs of all whole‐brain analyses, along with the data and analysis scripts for all statistical analyses, have been uploaded and made publicly available in a data repository at https://osf.io/y8t57/ (Open Science Framework; McDermott, 2020).

## 3 RESULTS

### 3.1 Behavioral data

**FIGURE 3:**
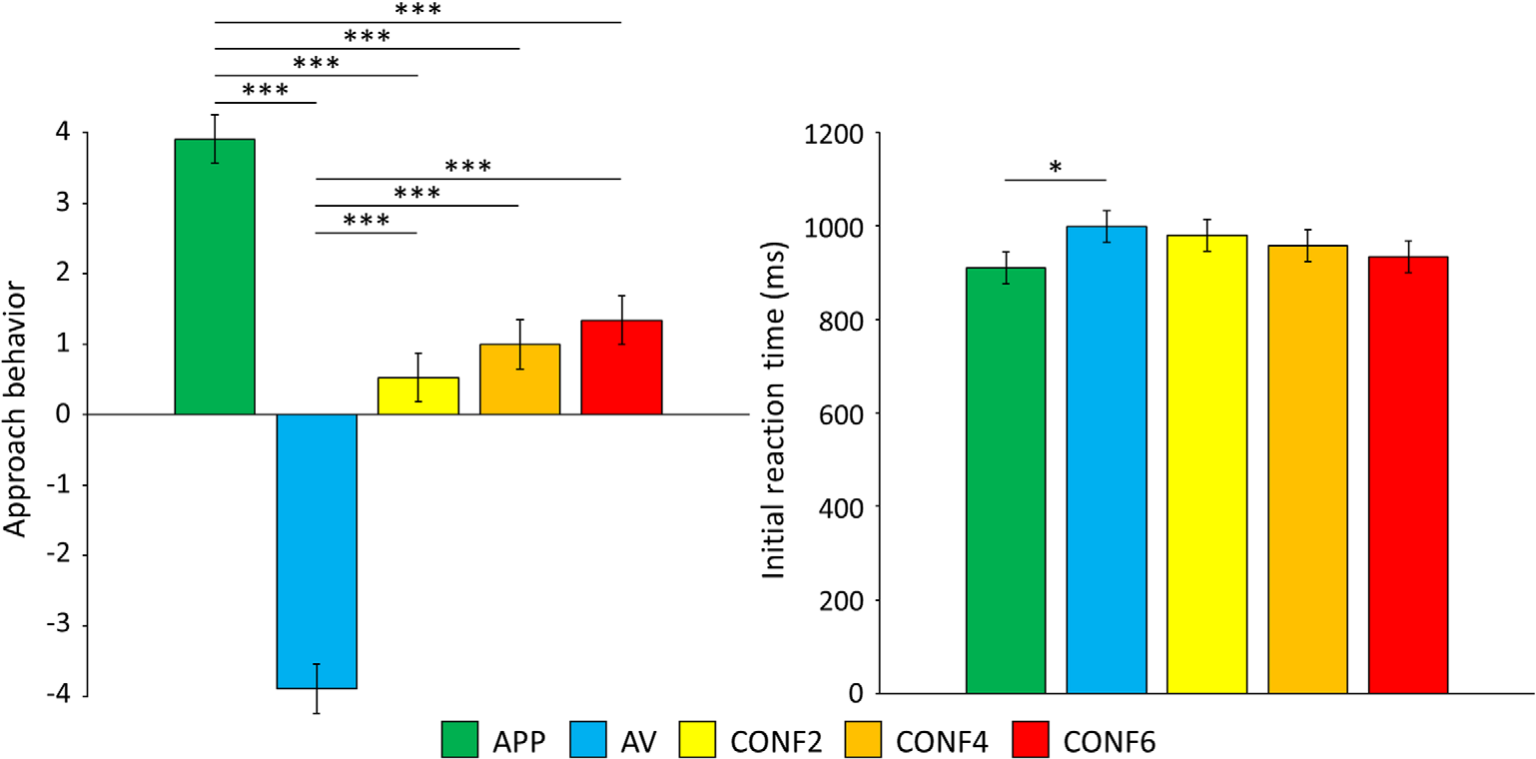
Approach‐avoidance conflict task behavioral results. Bar graphs depict group means (error bars depict ±1 standard error of the mean) for the main effect of condition for approach behavior and initial reaction time (RT) across time points. The color legend for each task condition is shown above (approach reward, APP; avoid threat, AV; conflict with 2‐cent reward, CONF2; conflict with 4‐cent reward, CONF4; conflict with 6‐cent reward; CONF6). Pairwise comparisons are denoted with “*” if significant at *p* <.05 or “***” if significant at *p* <.001. There was a significant main effect of time for reaction time (*p* = .016) but not for approach behavior. There were no significant condition‐by‐time interactions for either measure. [Left] Approach behavior (*N* = 30) was measured by the avatar's end position on the runway in relation to the negative outcome and/or reward, and this ranged from −4 (full avoidance from the negative outcome and/or reward) to +4 (full approach to the negative outcome and/or reward). [Right] Reaction time (RT; *N* = 27) was defined as when participants initially moved the joystick during the decision‐making phase of the task. Due to a software error in the joystick configuration, RT data were unavailable for three subjects

The mean test–retest period was 17.10 days (*SD* = 5.18; Range: 11–32). Time of day when scans were completed did not statistically differ between time points (*p* = .94). Bar graphs depicting means and standard errors for significant main effects of decision‐making condition on behavioral data are shown in Figure 3. ICCs for behavioral data are listed in Table 1. Means and standard deviations of approach behavior and RT for each individual condition and time point are provided in Table S1.

For approach behavior, 5 × 2 within‐subjects ANOVAs revealed a significant main effect of condition [*F*(4, 290) = 65.70, *p* <.001]. The main effect of time and the interaction between condition and time were both nonsignificant (*p*'s >.66). Follow‐up pairwise comparisons of the main effect of condition showed significant differences with increased approach behavior for APP versus AV trials, for APP versus all CONF trials, and for all CONF trials versus AV trials (all *p*'s <.001; Figure 3). Though there was a pattern of increasing approach behavior with increasing reward, approach behavior did not significantly differ across conflict conditions (all *p*'s >.46). For analyses of test–retest reliability, approach behavior was found to have excellent reliability (ICCs ≥.75) for all individual CONF trial types and when averaged.

For RT data (*N* = 27), 5 × 2 within‐subjects ANOVAs showed significant main effects of condition [*F*(4, 234) = 3.25, *p* = .016] and time [*F*(1, 234) = 30.37, *p* < .001]. The condition‐by‐time interaction was nonsignificant (*p* = .90). Follow‐up pairwise comparisons of the main effect of condition showed faster RT for APP compared to AV (Figure 3; *p* = .013), but there were no significant differences for RT between any of the other conditions (all *p*'s >.09). Regarding, the main effect of time, this was such that participants' RT was significantly faster at T2 compared to T1 regardless of condition. For analyses of test–retest reliability, RT had fair reliability (ICCs .4–.59) for all trial types (Table 1). This was the case for individual trial types, when averaged across CONF and NONCONF trial types, and when averaging across all trial types.

### 3.2 ROI data

**FIGURE 4:**
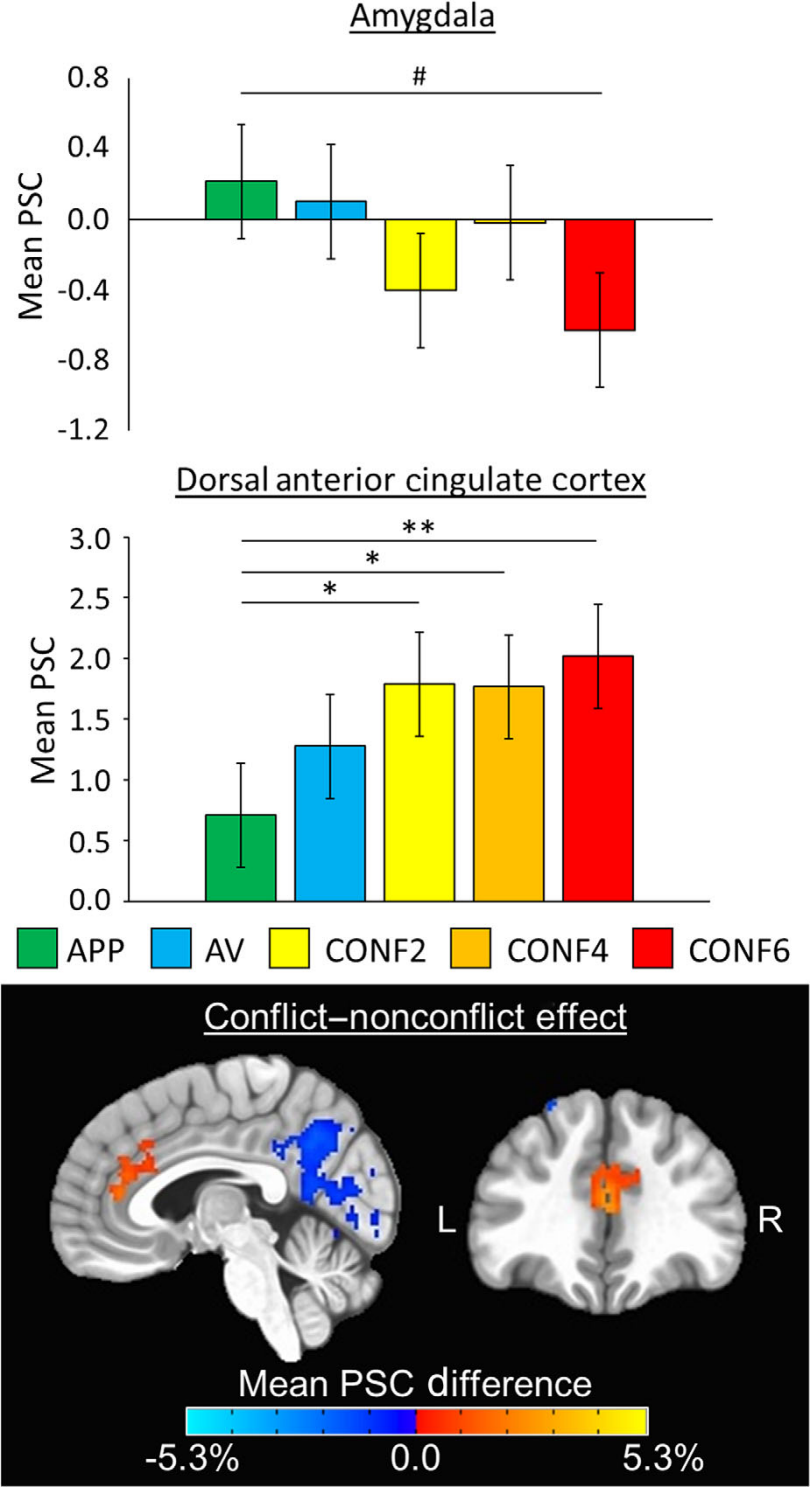
Decision‐making phase ROI and whole‐brain results. (Top) Bar graphs depict group mean percent signal change (PSC) data (error bars depict ±1 standard error of the mean) for the main effect of condition during decision‐making for both amygdala (*p* = .0499) and dorsal anterior cingulate cortex (*p* = .0018) composite regions of interest (ROIs) across time points. The color legend for each task condition is above (approach reward, APP; avoid threat, AV; conflict with 2‐cent reward, CONF2; conflict with 4‐cent reward, CONF4; conflict with 6‐cent reward; CONF6). Pairwise comparisons are denoted with ^#^ if marginally significant at *p* <.10, “*” if significant at *p* <.05, or “**” if significant at *p* <.0083. Significant main effects of time were found in amygdala, striatum, and anterior insula ROIs (all *p*'s ≤.004), but there were no significant condition‐by‐time interactions. (Bottom) Whole‐brain ANOVA *F*‐maps depicting the main effect of conflict decision‐making (i.e., comparing conflict (CONF2, CONF4, CONF6) to nonconflict (APP, AV) trials) across time points overlaid on the MNI152_T1_2009c T1‐weighted anatomical template brain in neurological orientation (i.e., left is left). Maps are thresholded at *p* <.01 and cluster‐corrected at 468 voxels based on multiple comparisons correction (*α* <.05, corrected). Color‐scheme for task‐related activation is such that red is greater PSC for conflict trials and blue is greater PSC for nonconflict trials (see color bars). Montreal Neurological Institute (MNI) coordinates for each slice displayed are as such: sagittal (*x* = 5) and coronal (*y* = 37)

**FIGURE 5:**
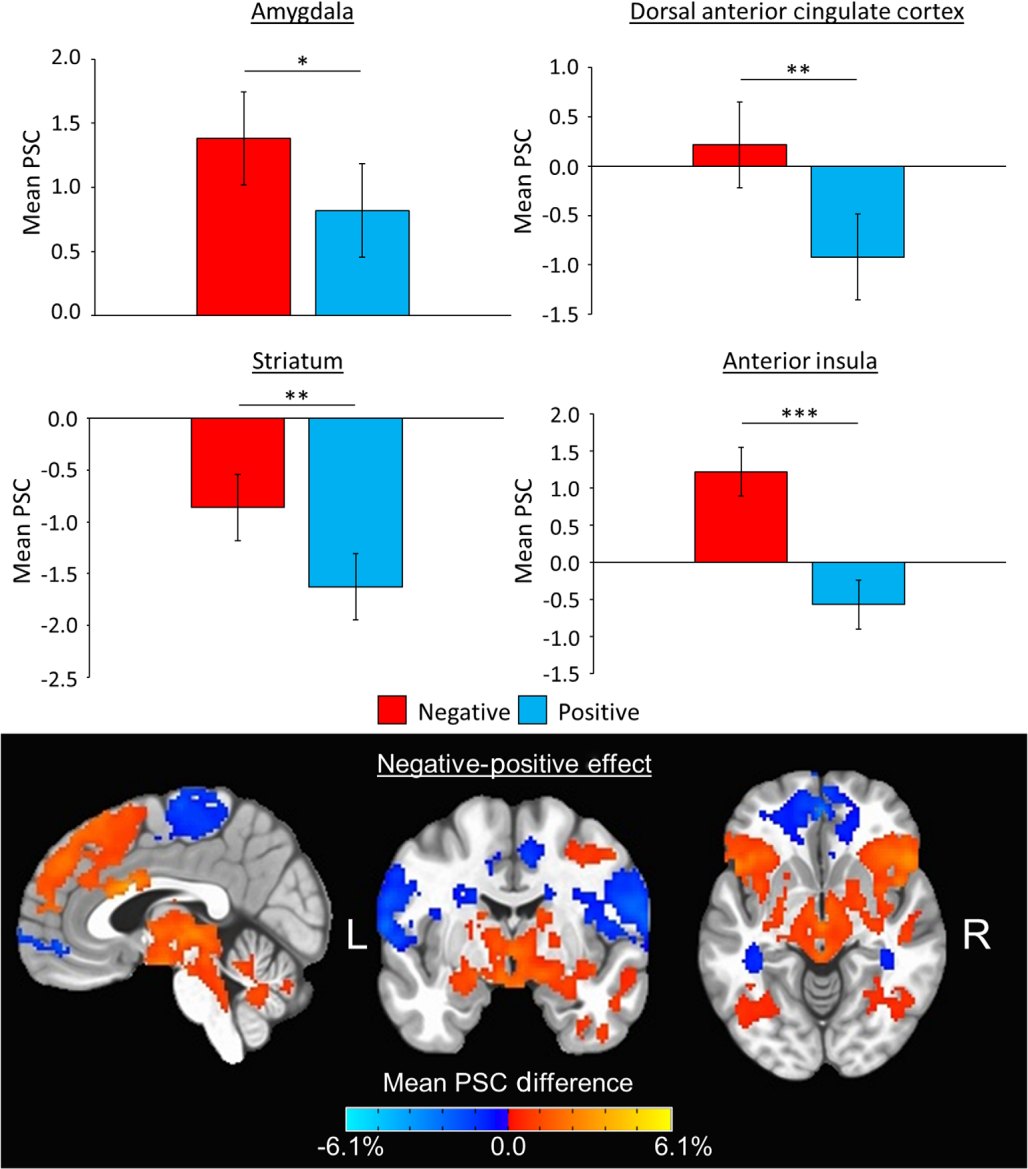
Affective outcome phase ROI and whole‐brain results. (Top) Bar graphs depict group mean percent signal change (PSC) data (error bars depict ±1 standard error of the mean) for the main effect of outcome for amygdala (*p* = .028), dorsal anterior cingulate cortex (*p* = .006), striatum (*p* = .0012), and anterior insula (*p* < .001) composite regions of interest (ROIs) across time points. The color legend for each task condition is above. Pairwise comparisons are denoted with “*” if significant at *p* <.05, “**” if significant at *p* <.0083, or “***” if significant at *p* <.001. Significant main effects of time were found in amygdala, left dorsolateral prefrontal cortex, and right dorsolateral prefrontal cortex ROIs (all *p*'s ≤.005), but there were no significant condition‐by‐time interactions. (Bottom) Whole‐brain ANOVA *F*‐maps depicting the main effect of outcomes (i.e., comparing negative to positive trials) across time points overlaid on the MNI152_T1_2009c T1‐weighted anatomical template brain in neurological orientation (i.e., left is left). Maps are thresholded at *p* <.01 and cluster‐corrected at 591 voxels based on multiple comparisons correction (*α* <.05, corrected). Color‐scheme for task‐related activation is such that red is greater PSC for negative trials and blue is greater PSC for positive trials (see color bars). Montreal Neurological Institute (MNI) coordinates for each slice displayed are as such: sagittal (*x* = 3), coronal (*y* = −5), and axial (*z* = −7)

Bar graphs depicting means and standard errors for significant main effects of condition on composite ROI data for decision‐making and outcome phases are shown in Figures 4 and 5, respectively. Means and standard deviations for extracted PSC for each of the six composite ROIs (i.e., amygdala, dACC, striatum, left dlPFC, right dlPFC, and anterior insula), for all contrasts and at both time points, are provided in Table S1 (decision‐making phase) and Table S2 (outcome phase and reward phase). Test–retest reliability ICCs for composite ROIs are listed in Table 2. ICCs for composite ROI subregions are listed in Table S3. Additionally, supplementary analyses showed that test–retest interval impacted reliability for only a subset of the ROIs for one of the contrasts (i.e., negative outcomes; Table S4), and higher PSC did not consistently relate to greater reliability (Table S5).

#### 3.2.1 Decision‐making phase

During the decision‐making phase, 5 × 2 within‐subjects ANOVAs revealed a significant main effect of condition in the dACC ROI [*F*(4, 261) = 4.41; *p* = .0018]. Additionally, there was a main effect of condition in the amygdala ROI [*F*(4, 261) = 2.41; *p* = .0499] that met our reporting threshold of *p* <.05. For dACC, follow‐up pairwise comparisons between conditions showed that mean PSC was significantly higher for decision‐making during each CONF trial type compared to APP trials (CONF2, *p* = .021; CONF4, *p* = .025; CONF6, *p* = .0023; Figure 4). For amygdala, follow‐up pairwise comparisons between conditions were all nonsignificant (all *p*'s >.073), but the pattern of findings showed a trend toward decreased mean PSC for CONF trials compared to NONCONF trials (*p* = .073 for APP compared to CONF6; Figure 4). There were significant main effects of time in the amygdala [*F*(1, 261) = 16.81, *p* <.001], striatum [*F*(1, 261) = 8.49, *p* = .004], and anterior insula [*F*(1, 261) = 25.85, *p* < .001] ROIs. These main effects of time were all such that mean PSC was significantly lower at T2 compared to T1, regardless of condition. There were no significant condition‐by‐time interactions during decision‐making for any of the six composite ROIs (*p*'s >.46).

For test–retest reliability analyses of the decision‐making phase (Table 1), individual PSC values during APP trials showed fair reliability in the dACC composite ROI. During AV trials, reliability was good in the left dlPFC ROI and fair in the dACC and right dlPFC ROIs. During CONF2 trials, reliability was fair in the dACC and right dlPFC ROIs. During CONF6 trials, reliability was fair in the dACC ROI. During averaged CONF trials, reliability was fair in the dACC, striatum, and left dlPFC ROIs. During averaged NONCONF trials, reliability was fair in the dACC, left dlPFC, and right dlPFC ROIs. For ROI data averaged across all trials, reliability was good in dACC and fair in striatum and left dlPFC. Supplementary analyses of test–retest reliability demonstrated that three ROI subregions showed good reliability during the decision making phase: left area 46 (AV trials), right pregenual dACC (CONF trials), and left dorsal area 9/46 (CONF trials; Supplementary Table 3).

#### 3.2.2 Outcome phase

During the outcome phase, 2 × 2 within‐subjects ANOVAs revealed significant main effects of condition in dACC [*F*(1, 87) = 7.76, *p* = .006], striatum [*F*(1, 87) = 11.28, *p* = .0012], and anterior insula [*F*(1, 87) = 33.67, *p* < .001] ROIs. Additionally, there was a main effect of condition in the amygdala ROI [*F*(1, 87) = 5.02, *p* = .028] that met our reporting threshold of *p* <.05. These main effects of condition were all such that mean PSC was higher for negative compared to positive outcomes, regardless of ROI (Figure 5). Significant main effects of time were found in amygdala [*F*(1, 87) = 10.58, *p* = .002], left dlPFC [*F*(1, 87) = 8.20, *p* = .005], and right dlPFC [*F*(1, 87) = 21.68, *p* = .003] ROIs. These main effects of time were all such that mean PSC in each of these ROIs were significantly lower at T2 compared to T1, regardless of condition. There were no significant condition‐by‐time interactions during outcomes for any of the six composite ROIs (*p*'s >.24).

For test–retest reliability analyses of the outcome phase (Table 2), individual PSC values showed good reliability for amygdala, dACC, striatum, and left dlPFC ROIs during negative outcomes. Meanwhile, reliability was fair for right dlPFC and anterior insula ROIs during negative outcomes. For positive outcomes, reliability was good for the striatum ROI and fair for the amygdala ROI. Supplementary analyses demonstrated one ROI subregion with excellent reliability during the outcome phase (Table S3), and this was the left nucleus accumbens during negative outcomes.

#### 3.2.3 ROI data: Reward phase

During the reward phase, 2 × 2 within‐subjects ANOVAs revealed no significant main effects of condition (all *p*'s >.33), no significant main effects of time (all *p*'s >.06), and no significant condition‐by‐time interactions (all *p*'s >.35). For test–retest reliability analyses of the reward phase (Table 2), individual PSC values showed reliability was fair for both striatum and dACC ROIs during the “reward” condition. Reliability was poor for all ROIs during the “no reward” condition. Supplementary analyses demonstrated two ROI subregions showed good reliability during the reward phase, and these were the left nucleus accumbens and left dorsal agranular insula during the “reward” condition (Table S3).

### 3.3 Whole‐brain data

Results from whole‐brain voxel‐wise analyses showed no significant condition‐by‐time interactions for any of the three models based on the *p* <.01 threshold, corrected for multiple comparisons at *α* <.05. For the decision‐making ANOVA, there were a total of five significant clusters for effect of condition (*p* <.01, corrected; Figure 4). One of the significant clusters for the effect of condition was located in dACC (563 voxels; peak MNI coordinates: 1, 39, 15), and this showed activity was higher during CONF compared to NONCONF trials. Three of these clusters peaked in regions of the default mode network (Raichle et al., 2001), including posterior cingulate cortex (6,869 voxels; MNI: 1, −65, 51), left lateral parietal lobule (1,208 voxels; MNI: −47, −67, 47), and left parahippocampal gyrus (754 voxels; peak MNI: −31, −41, −13). All of these showed lower activity during CONF compared to NONCONF trials. The last cluster peaked in left superior frontal gyrus (756 voxels; MNI: −35, 21, 57), and this also showed lower activity during CONF compared to NONCONF trials. There were no significant clusters for effect of time for the decision‐making phase.

For the outcome ANOVA, there was a total of six significant clusters for effects of condition (*p* <.01, corrected; Figure 5), which spanned several large areas of the brain. For example, one of the largest clusters for effect of condition was 11,650 voxels and showed greater activity for negative compared to positive outcomes. This cluster included right amygdala (MNI: 27, 7, −25), right anterior insula, bilateral striatum, and right middle frontal gyrus. Meanwhile, the largest cluster was 16,933 voxels and showed less activity for negative compared to positive outcomes. This cluster encompassed orbitofrontal cortex (MNI: −1, 49, −9), posterior insula, and superior temporal gyrus. Other clusters and MNI peaks that showed greater activity for negative compared to positive outcomes included dACC (3,064 voxels; MNI: 1, 13, 25), left anterior insula (3,041 voxels; MNI: −31, 17, −19), right temporal pole (2,616 voxels; MNI: 47, 5, −31), and left temporoparietal junction (1,838 voxels; MNI: −59, −49, 27). There were also three significant clusters for effects of time (*p* <.01, corrected), which peaked in the left primary visual cortex (2,086 voxels; MNI: −13, −59, 3), right superior parietal lobule (1,855 voxels; MNI: 7. −67, 63), and left superior parietal lobule (1,580 voxels; MNI: −7, −67, 65). All of the these showed less activation at T2 compared to T1. For the reward ANOVA, there were no significant clusters for effects of condition or time. All whole‐brain ANOVA maps, which include individual contrast maps for further examination, have been provided for public access in the online data repository for this study (McDermott, 2020).

**FIGURE 6:**
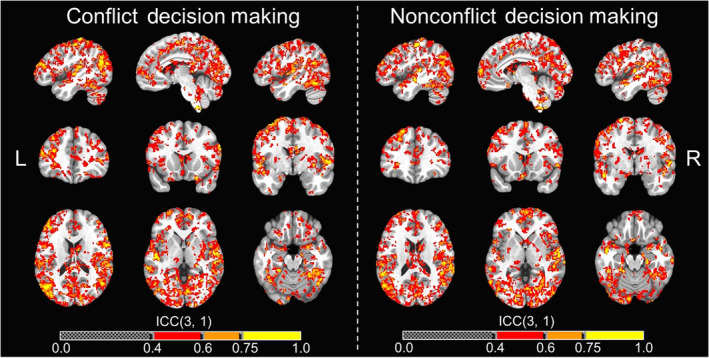
Neural activation whole‐brain voxel‐wise ICC maps for decision‐making phase. Whole‐brain voxel‐wise intraclass correlation coefficient (ICC) maps for the conflict and nonconflict decision‐making contrasts across T1 and T2 overlaid on the MNI152_T1_2009c T1‐weighted anatomical template brain in neurological orientation (i.e., left is left). Color‐scheme for ICCs (see color bars) that were at least fair goes as such: red = fair reliability (ICCs = .4–.59), good reliability (ICCs = .60–.74), yellow = excellent reliability (ICCs = .75 or greater). Montreal Neurological Institute (MNI) coordinates for each slice (displayed from left to right) are as such: sagittal (top; *x* = −46, −6, 46), coronal (middle; *y* = 35, 15, −3), axial (bottom; *z* = 18, 0, −19)

**FIGURE 7:**
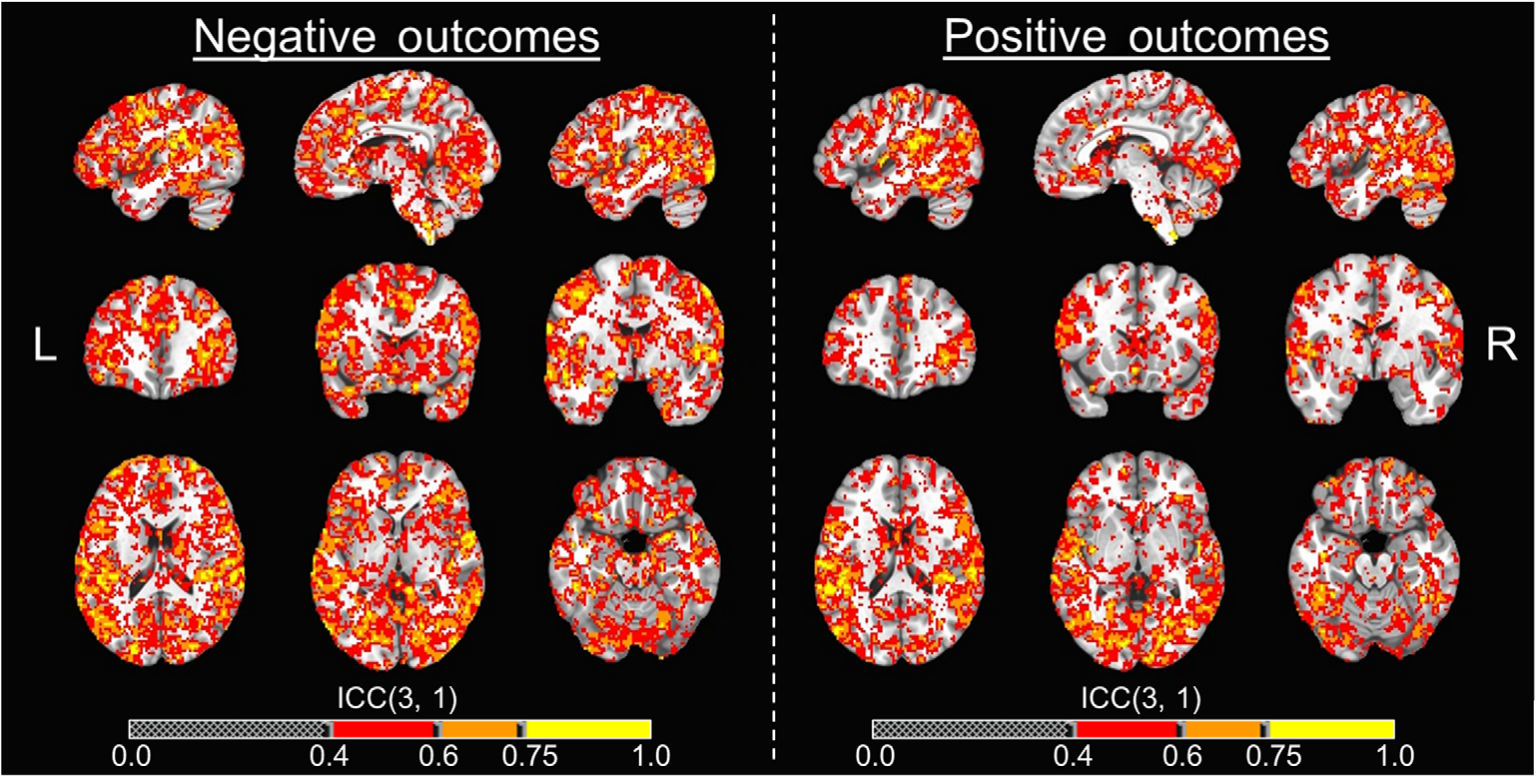
Neural activation whole‐brain voxel‐wise ICC maps for outcome phase. Whole‐brain voxel‐wise intraclass correlation coefficient (ICC) maps for the negative and positive affective outcome contrasts across T1 and T2 overlaid on the MNI152_T1_2009c T1‐weighted anatomical template brain in neurological orientation (i.e., left is left). Color‐scheme for ICCs (see color bars) that were at least fair goes as such: red = fair reliability (ICCs = .4–.59), good reliability (ICCs = .60–.74), yellow = excellent reliability (ICCs = 0.75 or greater). Montreal Neurological Institute (MNI) coordinates for each slice (displayed from left to right) are as such: sagittal (top; *x* = −46, −6, 46), coronal (middle; *y* = 35, 15, −3), axial (bottom; *z* = 18, 0, −19)

Whole‐brain voxel‐wise ICCs for each of the six contrasts examined showed clusters with at least fair reliability. Based on the total number of voxels that were considered fair (ICCs = .4–.59), good (ICCs = .6–.74), or excellent (ICCs ≥.75) for each contrast, the rank order in terms of reliability for each contrast goes as such: negative outcomes (110,115 voxels), positive outcomes (80,076 voxels), CONF decision‐making (64,944 voxels), NONCONF decision‐making (64,675 voxels), “reward” (47,720 voxels), and “no reward” (44,353 voxels). Whole‐brain ICC maps for CONF and NONCONF decision‐making trials are displayed in Figure 6, and whole‐brain ICC maps for negative and positive outcomes are displayed in Figure 7. All whole‐brain ICC maps for all individual contrasts have also been provided for public access in the study's online data repository (McDermott, 2020).

## 4 DISCUSSION

In the present study, we conducted replication and test–retest reliability analyses of behavioral and neural responses during an approach‐avoidance conflict task (Aupperle et al., 2015). Behavioral responses on the task were, for the most part, replicable and exhibited fair to excellent reliability (ICCs ≥.40). Neural responses to the task partly replicated previous findings by identifying significant condition effects in several a priori ROIs, including dACC for conflict decision‐making (vs. both nonconflict conditions) and amygdala, insula, striatum, and dACC for negative affective outcomes (vs. positive). There were also several ROIs for which activation was found to have fair (29.6% of ROIs) to good (11.1% of ROIs) reliability for individual contrasts. Thus, findings support the use of this approach‐avoidance conflict task in future research and provide guidance on which variables may be particularly useful for studies of individual differences and/or intervention effects.

Overall, behavioral responses to the task were very similar to those reported previously, with the presence of conflict significantly modulating approach behavior and/or RT in the expected directions (Aupperle et al., 2011, 2015). While RT data demonstrated significant changes as a result of practice (i.e., faster responses at T2), ICC calculations (which accounted for practice effects) for RT indicated fair reliability. On the other hand, approach behavior during conflict trials had excellent reliability. These findings are important considering recent work suggesting that computer‐based behavioral tasks often exhibit poor reliability (Hedge et al., 2018). Future applications of the approach‐avoidance conflict task could utilize approach behavior as a metric and place high confidence in both its replicability across studies/samples and its reliability over time for individual participants.

Neural data from this task partially replicated what was reported for whole‐brain analyses in the previous fMRI study with this task (Aupperle et al., 2015). During decision‐making, the previous finding that dACC activity is modulated by conflict was successfully replicated in both ROI and whole‐brain analyses, but previous findings that conflict modulates right dlPFC, anterior insula, and striatum activity were not replicated. These findings are consistent with propositions that the dACC plays a primary role in the processing of conflict, particularly during affective conflict (Braem et al., 2017). Interestingly, amygdala was modulated by conflict at our reporting threshold of *p* <.05, an effect that was not found in the prior study (Aupperle et al., 2015). However, the directionality of these results is perhaps counterintuitive, as amygdala was more active during approach‐reward trials compared to conflict trials. Though speculative, it is possible that this finding reflects a combination of (1) the general salience of an opportunity for reward during approach‐reward trials and (2) greater cognitive load to support decision‐making during the conflict trials resulting in the inhibition of amygdala activity (Bissonette & Roesch, 2015). Test–retest reliability analyses of neural responses during the decision‐making phase found that 20.0% of the ROIs included had at least fair reliability (ICCs ≥.40; average ICC for ROIs = 0.242; range of ICCs: −.18 to .65). Note that dACC had fair reliability across 4 out of 5 decision‐making conditions, with good reliability when averaged across all conditions (ICC = .61). While these reliability estimates would all ideally be in the good or excellent range, the findings from this study indicate that dACC activity is both reproducible and reliable enough to support its application in future studies of decision‐making with this task, albeit with caution.

Neural data examined during the outcome phase of the approach‐avoidance conflict task were broadly found to be both reproducible and reliable. Findings that amygdala, dACC, striatum, and anterior insula are significantly modulated by outcome valence replicated previous work (Aupperle et al., 2015), although previous findings of dlPFC modulation were not replicated. Whole‐brain analyses also identified differences between outcome conditions across both time points in bilateral amygdala, dACC, striatum, and anterior insula. Reliability analyses found that 66.7% of the ROIs included had at least fair reliability (ICCs ≥.40; average ICC for ROIs = 0.483; range of ICCs: .15–.69). This included good reliability for amygdala, dACC, striatum, and left dlPFC ROIs. These ICC estimates are better than expected, given that prior work examining the test–retest reliability of amygdala activation during passive viewing of affective stimuli report poor to fair ICCs (Hassel et al., 2020; Lois et al., 2018; McDermott et al., 2020). It is possible that the active processing of these stimuli within a decision‐making context might require more engagement of participants, which could therefore lead to more robust and reliable neural activity. In addition, the combination of both visual and auditory stimuli may also lead to increased attentional engagement or salience processing. The decision‐making context may also increase the ecological validity of the task, which has been suggested to potentially improve reliability (Sonkusare, Breakspear, & Guo, 2019). Regardless of the reasons for the difference of this task compared to others, future applications of this task can be relatively confident in using neural data from the outcome phase.

For the reward phase of the task, note that although we hypothesized that reward phase conditions would significantly modulate regions such as striatum or dlPFC (based on previous research with other tasks [Wang et al., 2016]), this nonsignificant effect was a replication of the prior study's findings (Aupperle et al., 2015). This lack of an effect may be due to the timing of the task and how reward outcomes phases are presented immediately after the highly salient affective outcomes. Reliability analyses showed that the reward phase was the least reliable task phase with only 16.7% of ROIs showing at least fair reliability (ICCs ≥.40; average ICC of ROIs = 0.232/range of ICCs: .13–.58). It is therefore suggested that, unless there are modifications to the task, future applications should focus on decision‐making or outcome phases rather than the reward phase.

The average reliability coefficient found in the present study (average ICC: .33) is generally consistent with the review of findings detailed by Elliott et al. (2020; average ICC .397). However, we disagree with the outright notion that ROI‐based approaches to fMRI data cannot yield reliable results. The process of refining and optimizing measurement approaches to yield data with improved reliability and validity is a necessary approach within any field of science, including human neuroimaging (Poldrack et al., 2016). Broad statements of fMRI methodology as being inherently unreliable could lead to problematic conclusions, as evidenced in a recent popular media article (Cohen, 2020) that states “every brain activity study you've ever read is wrong” when reporting on findings from the Elliott et al. (2020) meta‐analysis. As these meta‐analytic findings are appropriately concerning though, fMRI studies should focus on improving reliability. Note that alternative data analytic approaches present another possible solution to yield more reliable fMRI findings (e.g., multivariate pattern analysis; Kragel et al., 2020), but the differences in the interpretation and potential applicability of these approaches should be considered when using these approaches (Hebart & Baker, 2018). While guidelines suggest that ICCs ≥.60 are optimal for reliability (Fleiss, 1986), the reliability cutoff for neuroimaging approaches, such as fMRI, may need to be lowered (e.g., ICCs ≥.40) as these approaches provide information that cannot be obtained with other measurement tools. Last, other confounding factors could alter the measurement between time points that are not attributable to measurement error but also are not consistent across the group (e.g., the current mental state of the individual). While others have suggested that such confounding factors should be controlled for in the study design (Bennett & Miller, 2010), this is not always possible or even ideal, such as when the task is focused on domains of processing that are intricately linked with one's mental state. The results herein suggest that fMRI tasks can be designed to have sufficient reliability, and specific ways of quantifying brain activity can be identified that result in sufficient reliability.

There are several limitations to consider for the present study. The sample size was perhaps smaller than optimal (*N* = 30), though it exceeds the recommended *N* = 20 for detecting ICCs of .60 with 90% power for two observations (Bujang & Baharum, 2017). Note also that there were several participants excluded from the sample due to excessive motion or technical errors. Future work with this task would benefit from efforts at minimizing these issues. In addition, it is unknown whether the ICCs identified for healthy participants in this study would remain consistent for clinical populations. Examining reliability in larger and more diverse samples could further support generalizability. Next, we used a composite ROI approach in order to reduce the total number of statistical comparisons and to account for individual variability in peak voxels of activation. However, this approach could have removed important variability across subregions. As a partial remedy for this, we included ICC estimates for each of the composite ROI subregions. In addition, the Brainnetome atlas utilized for ROI mapping in this study is one of many standardized atlases, and the specific atlas utilized could affect the results. Future work might examine reliability using alternative ROI atlases, and the publicly available whole‐brain ICC maps from this study could be used for this purpose. Lastly, although each individual's data were normalized in standard MNI space, there could have been individual anatomical variability that would affect the quality of fit for each ROI. While this approach used a standardized atlas to support generalizability of these findings to other samples, future reliability studies might consider using individualized approaches for mapping anatomical structures.

In conclusion, the present study demonstrated evidence of neural and behavioral mechanisms of approach‐avoidance conflict that are both replicable and reliable during fMRI. Note that while many of the neural responses examined were not found to be reliable, these findings provide value to future research by ruling out the use of these unreliable responses in future work. Overall, this study characterized several specific behavioral variables and brain ROIs that may be optimal to use in future applications of this approach‐avoidance conflict task. For example, such variables could be of interest in examining individual differences (e.g., in relation to clinical symptom severity or prediction of treatment outcome) or for examining mechanisms of intervention effects (i.e., in conjunction with randomized clinical trials for anxiety disorders [Santiago et al., 2020]). Additionally, this study presents a framework for how other task paradigms might comprehensively examine these mechanisms in order to identify findings that are both reproducible and reliable.

## CONFLICT OF INTEREST

The authors have no conflict of interest to disclose.

## Supporting information


**Appendix S1:** Supporting Information

## Data Availability

All data for this study were collected at the Laureate Institute for Brain Research, and the research protocols used were approved by the Western Institutional Review Board. Final neuroimaging analysis output files have been uploaded and made publicly available in a data repository at https://osf.io/y8t57/ (McDermott, 2020; DOI: 10.17605/OSF.IO/Y8T57). Additionally, the region of interest files used for analyses as well as the final analysis scripts have all been uploaded and made publicly available in this same repository. Raw data that were used to conduct these analyses can be made available to those who wish to replicate our analyses upon request to the authors.
