# Supplementary material for: Test–retest reliability of approach‐avoidance conflict decision‐making during functional magnetic resonance imaging in healthy adults
Source: Hum Brain Mapp. 2021 Mar 2;42(8):2347–61. doi: 10.1002/hbm.25371 (PMC8090786; doi:10.1002/hbm.25371)
Supplement: Supplementary file 1 — Appendix S1: Supporting Information [file HBM-42-2347-s001.docx]

**Supplementary Material**

1. *Supplemental Methods and Results*

*1.1. Expanded experimental paradigm and stimuli description*

The approach-avoidance conflict task used in this study had three phases: (1) decision-making, (2) affective outcome, and (3) monetary reward (Aupperle et al., 2015). During the decision-making phase, participants were presented with a runway that had pictures on each side to represent two possible outcomes. The possible outcomes included both an image (i.e., sun or cloud) indicative of an affective stimulus and a level of monetary reward (i.e., 0, 2, 4, or 6 in United States’ cents) on each side. The image of a sun indicated a positively valenced stimulus outcome, and a cloud indicated a negatively valenced stimulus outcome. Level of reward was indicated by the amount of red ink filling a rectangular meter adjacent to the sun or cloud. Participants used a joystick to move an avatar on the runway to indicate their preference for the potential outcomes. At the end of the decision phase, the avatar’s location corresponded to the probability of each of the two outcomes occurring, with each marker on the runway varying the probability of the outcomes by 10%. If the participant moved the avatar to the middle of the runway, there was a 50% chance of either outcome. The participant could raise the probability of an outcome to as high as 90% by moving the avatar all the way to one side. While participants could determine likelihood of either outcome, they could not determine the outcome with complete certainty. To control for potential influence of the avatar’s starting position on reaction time or decisions, the avatar starting position was counterbalanced across trials.

During the outcome phase, participants were presented with either positively valenced or negatively valenced pictures and sounds that were drawn from the International Affective Picture System (IAPS; Lang et al., 2008), the International Affective Digitized Sounds (IADS; Bradley and Lang, 1999), and other public domain audio files. During the reward phase, participants were given 0, 2, 4, or 6 cents [unlike previous work with this same task that used points (Aupperle et al., 2015)], and different tones played depending if a reward was given or not. Given all of these parameters, the task was designed with three trial types based on the expected behavioral motivation during decision-making in relation to possible outcomes and rewards. The first trial type was ‘Approach-reward’ (APP), in which a reward of 2 cents was offered for a positive outcome on one side and 0 cents for positive outcome on the other side. This was intended to motivate participants to move the avatar toward the positive image/sound outcome that also had a reward. The second trial type was ‘Avoid-threat’ (AV), in which a reward of 0 cents was offered for both a positive and negative outcome on each side. This was intended to motivate participants to move the avatar away from the negative image/sound outcome. The third trial type was ‘Conflict’ (CONF), in which 2 (CONF2), 4 (CONF4), or 6 (CONF6) cents were offered for the negative outcome while 0 cents were offered for the positive outcome. These trials intended to produce “approach-avoidance conflict” in participants such that a decision to approach the reward also presented greater risk of a negative image/sound outcome. The varying levels of reward sought to increase motivation for participants to approach the reward despite the increased risk of a negative outcome.

The task used an event-related design with a total of 90 trials (18 of each trial type: AV, APP, CONF2, CONF4, CONF6) over three fMRI scans (i.e., 30 trials per scan). The stimulus presentation software used was PsychoPy (Version 1.84.2). Prior to performing the task, participants received detailed instructions and completed four practice trials to ensure sufficient understanding. Practice stimuli were included in the sample of stimuli during the main task. The full sample of affective images and sounds was the same across the fMRI scans at each time point. However, the individual set of affective stimuli differed for each of the three fMRI runs, and the block order was randomized for each time point. Additionally, note that the specific outcomes individuals were exposed to differed based on the choices they made during the decision-making phase. Each trial of the task provided participants with 4 seconds during the decision-making phase, 6 seconds of affective stimuli outcome presentation, 2 seconds of reward presentation, and an intertrial interval of 1-7 seconds (mean = 4 seconds). Each individual scan lasted 480 seconds (i.e., 8 minutes), which is a total of 1,440 seconds (i.e., 24 minutes) across the three scans. The task was divided across three scans to provide participants the opportunity to rest in-between each run and therefore maintain alertness during task performance. Task performance was measured through (1) approach behavior and (2) initial reaction time. Approach behavior was measured by the avatar’s end position on the runway in relation to the negative outcome and/or reward, and this ranged from -4 (full avoidance from the negative outcome and/or reward) to +4 (full approach to the negative outcome and/or reward). Reaction time (RT) was defined as when participants initially moved the joystick during the decision-making phase (i.e., first avatar position change). Approach behavior and RT were calculated for each participant and averaged by trial type. Due to a software error in the joystick configuration, RT data were unavailable for three subjects.

*1.2. Reliability Analyses of Composite ROI Subregions*

Mean PSC data were extracted from voxels within individual composite ROI subregions. The means and standard deviations for these data for each contrast and time point are provided in Supplementary Table 1 (decision-making phase) and Supplementary Table 2 (outcome and reward phases). Supplementary analyses of test-retest reliability were conducted by estimating ICC(3, 1) for individual mean PSC values across T1 and T2 separately for all composite ROI subregions. These are provided in Supplementary Table 3. Note that these supplementary analyses did not separate conflict contrasts by level of reward to simplify interpretation.

*1.3.* *Exploratory Correlations Using ROI Subregion Data*

Lastly, we conducted additional supplementary analyses of ROI data to examine the following questions about reliability: (1) does the test-retest interval (i.e., days between scans) relate to test-retest reliability? and (2) does higher level of absolute mean PSC at T1 relate to greater test-retest reliability estimates? For question 1, we conducted Pearson’s correlations between test-retest interval and change in mean PSC across time within the six composite ROIs for three contrasts of interest (i.e., conflict decision-making, negative outcomes, reward). As a follow-up analysis, we then calculated ICCs using the residuals of correlations between test-retest interval and mean PSC at each individual time point to see if this improved our reliability estimates. The full results of these analyses are provided in Supplementary Table 4. For question 2, we conducted Pearson’s correlations between absolute activation from 22 ROI subregions at T1 related to reliability in those 22 ROIs (i.e., ICCs). These were conducted for each individual contrast during each of the task phases for a total of seven contrasts. As above, these analyses did not separate conflict contrasts by level of reward to simplify interpretation. The full results of these analyses are provided in Supplementary Table 5.

Regarding question 1, during conflict decision-making and reward contrasts, none of these correlations were significant between test-retest interval and change in mean PSC (all p’s > .19). During negative outcomes, there were three significant negative correlations (all p’s < .005) between test-retest interval and change in mean PSC across time in the dACC (r = -0.50), left dlPFC (r = -.54), and right dlPFC composite ROIs (r = -0.67). These correlations were such that the longer test-retest period between scans, data were less consistent in these three ROIs during negative outcomes. For each of the three ROIs that had a relationship with test-rest interval, reliability estimates were higher, and this was most evident in the left dlPFC (original ICC = .49 / adjusted ICC = .67). Thus, future work should consider accounting for test-retest interval if there is interest in examining mean PSC change in these specific ROIs during negative outcomes. However, other ROIs seem to be unaffected by test-retest interval. Regarding question 2, the results of these analyses were inconclusive as the correlational relationships varied by particular contrast and task phase. During the decision-making, these relationships were all in the positive direction and modest strength (*r*’s 0.37-0.45). During the outcome phase, these were in the opposing directions showing a negative correlational relationship for one contrast (negative outcomes; *r* = -0.53) and a positive correlational relationship for the other contrast (positive outcomes; *r* = 0.36). During the reward phase, there was a small positive relationship during ‘reward’ trials (*r* = 0.29) but no relationship during ‘no reward’ trials (*r* = 0.02). The overall Pearson’s r including data from all ROIs (i.e., 154 data points) for all trial types was *r* = .17.

**Supplementary Table 1. Means and Standard Deviations for Behavioral Measures and Composite ROI PSC Values during Decision-Making Phase**

|  | **Decision-making Trial Type** | | | | | | | | | | | | | | | |
| --- | --- | --- | --- | --- | --- | --- | --- | --- | --- | --- | --- | --- | --- | --- | --- | --- |
| **Behavioral**  **Measures** | Approach | | Avoid | | Conflict-2 | | Conflict-4 | | Conflict-6 | | Conflict-Averaged | | Non-conflict-  Averaged | | All-Averaged | |
|  | T1 | T2 | T1 | T2 | T1 | T2 | T1 | T2 | T1 | T2 | T1 | T2 | T1 | T2 | T1 | T2 |
| Approach  Behavior | 3.88  (0.19) | 3.86  (0.26) | -3.79  (0.53) | -3.86  (0.40) | 0.97  (3.27) | 0.37  (3.62) | 1.36  (3.42) | 0.90  (3.50) | 1.67  (3.27) | 1.26  (3.46) | 1.33  (3.24) | 0.84  (3.39) | - | - | - | - |
| Reaction  Time^@^ | 949  (152) | 866  (141) | 1028  (159) | 955  (208) | 1050  (286) | 923  (224) | 1014  (210) | 900  (207) | 976  (222) | 880  (187) | 1013  (227) | 901  (194) | 988  (138) | 911  (151) | 1003  (183) | 905  (165) |
| **Composite ROI** |  |  |  |  |  |  |  |  |  |  |  |  |  |  |  |  |
| Amygdala | 0.82  *(1.92)* | -0.39  *(2.24)* | 0.52  *(1.94)* | -0.31  *(1.68)* | 0.15  *(1.50)* | -0.95  *(2.30)* | 0.07  *(1.79)* | -0.11  *(1.79)* | -0.18  *(2.21)* | -1.07  *(2.11)* | 0.01  *(1.51)* | -0.70  *(1.64)* | 0.67  *(1.74)* | -0.35  *(1.79)* | 0.27  *(1.45)* | -0.57  *(1.63)* |
| Dorsal ACC | 1.12  *(2.44)* | 0.30  *(3.21)* | 1.20  *(2.40)* | 1.35  *(2.80)* | 1.90  *(2.59)* | 1.68  *(3.15)* | 1.90  *(2.42)* | 1.64  *(2.91)* | 2.31  *(2.46)* | 1.73  *(2.59)* | 2.02  *(1.95)* | 1.66  *(2.45)* | 1.16  *(2.33)* | 0.82  *(2.72)* | 1.69  *(2.03)* | 1.34  *(2.42)* |
| Striatum | 0.93  *(1.60)* | 0.57  *(1.94)* | 0.90  *(1.58)* | 0.98  *(1.46)* | 1.02  *(1.27)* | 0.47  *(1.55)* | 1.47  *(1.25)* | 0.78  *(1.65)* | 1.03  *(1.31)* | 0.53  *(1.47)* | 1.16  *(0.97)* | 0.59  *(1.18)* | 0.91  *(1.48)* | 0.78  *(1.58)* | 1.07  *(1.11)* | 0.67  *(1.25)* |
| Left  DLPFC | 1.05  *(1.49)* | 1.28  *(2.40)* | 1.21  *(1.20)* | 1.17  *(1.57)* | 0.97  *(1.23)* | 1.10  *(1.51)* | 1.27  *(1.60)* | 1.30  *(1.56)* | 1.03  *(1.54)* | 0.97  *(1.04)* | 1.08  *(1.20)* | 1.11  *(1.12)* | 1.13  *(1.21)* | 1.23  *(1.85)* | 1.10  *(1.14)* | 1.16  *(1.29)* |
| Right DLPFC | 1.65  *(1.59)* | 1.81  *(2.08)* | 1.68  *(1.32)* | 1.66  *(1.85)* | 1.56  *(1.58)* | 1.60  *(1.49)* | 1.85  *(1.37)* | 1.74  *(1.43)* | 1.53  *(1.40)* | 1.38  *(1.53)* | 1.63  *(1.18)* | 1.56  *(1.16)* | 1.66  *(1.29)* | 1.74  *(1.76)* | 1.65  *(1.10)* | 1.64  *(1.25)* |
| Anterior Insula | 1.60  *(2.27)* | 0.36  *(2.07)* | 2.11  *(2.35)* | 1.02  *(2.17)* | 2.13  *(1.67)* | 1.04  *(1.93)* | 2.30  *(1.69)* | 1.21  *(2.23)* | 2.28  *(2.11)* | 1.43  *(1.84)* | 2.21  *(1.46)* | 1.21  *(1.34)* | 1.85  *(2.07)* | 0.69  *(1.80)* | 2.08  *(1.59)* | 1.01  *(1.29)* |

Means and standard deviations (in italics) are listed for all ROIs for each contrast and time point. Initial reaction time ICC estimates included data from 27 out of the 30 participants and thus are denoted with a ^@^. Abbreviations: ROI=region of interest; PSC=percent signal change; DLPFC=dorsolateral prefrontal cortex.

**Supplementary Table 2. Means and Standard Deviations for Composite ROI PSC Values during Outcome and Reward Phases**

|  | **Outcome Phase** | | | | **Reward Phase** | | | |
| --- | --- | --- | --- | --- | --- | --- | --- | --- |
| **Composite ROI** | Negative | | Positive | | Reward | | No Reward | |
|  | T1 | T2 | T1 | T2 | T1 | T2 | T1 | T2 |
| Amygdala | 1.94  *(2.28)* | 0.83  *(2.42)* | 1.08  *(1.88)* | 0.56  *(2.24)* | 1.18  *(1.46)* | 0.73  *(2.49)* | 1.00  *(1.95)* | 0.75  *(1.98)* |
| Dorsal ACC | 0.61  *(3.49)* | -0.18  *(3.62)* | -0.60  *(1.96)* | -1.24  *(1.88)* | 0.02  *(2.13)* | -0.27  *(2.06)* | 0.27  *(1.49)* | -0.07  *(3.01)* |
| Striatum | -0.70  *(2.47)* | -1.03  *(2.34)* | -1.36  *(1.49)* | -1.89  *(1.25)* | 1.53  *(1.39)* | 1.14  *(1.41)* | 1.53  *(1.27)* | 1.45  *(1.40)* |
| Left  DLPFC | -1.04  *(2.19)* | -1.78  *(2.08)* | -1.35  *(1.34)* | -2.03  *(1.35)* | 0.96  *(1.20)* | 1.15  *(1.66)* | 0.81  *(1.21)* | 0.99  *(1.29)* |
| Right DLPFC | -0.52  *(2.44)* | -1.29  *(2.15)* | -0.81  *(1.71)* | -1.74  *(1.46)* | 0.82  *(1.71)* | 1.18  *(1.93)* | 0.93  *(1.57)* | 0.87  *(1.81)* |
| Anterior Insula | 1.28  *(2.29)* | 1.17  *(2.51)* | -0.58  *(1.90)* | -0.57  *(1.91)* | 1.17  *(2.10)* | 0.86  *(2.35)* | 1.73  *(2.30)* | 0.90  *(1.73)* |

Means and standard deviations (in italics) are listed for all ROIs for each contrast and time point. Abbreviations: ROI=region of interest; PSC=percent signal change; DLPFC=dorsolateral prefrontal cortex.

**Supplementary Table 3. ICCs for Brainnetome Composite ROI Subregions**

|  | Decision Making Phase | | | Outcome Phase | | Reward Phase | |
| --- | --- | --- | --- | --- | --- | --- | --- |
| ROI Subregions | *Approach* | *Avoid* | *Conflict-Averaged* | *Negative* | *Positive* | *Reward* | *No Reward* |
| **Amygdala** |  |  |  |  |  |  |  |
| *Left Lateral,*  *BN 211* | -0.06  *(-0.41-0.30)* | 0.33  *(-0.03-0.61)* | -0.05  *(-0.40-0.31)* | 0.74*  *(0.52-0.87)* | 0.47^#^  *(0.14-0.71)* | 0.29  *(-0.07-0.59)* | 0.58^#^  *(0.28-0.78)* |
| *Right Lateral,*  *BN 212* | 0.13  *(-0.24-0.46)* | 0.13  *(-0.24-0.46)* | 0.42^#^  *(0.07-0.67)* | 0.60*  *(0.31-0.79)* | 0.52^#^  *(0.20-0.74)* | 0.42^#^  *(0.08-0.68)* | 0.37  *(0.01-0.64)* |
| *Left Medial,*  *BN 213* | 0.10  *(-0.27-0.44)* | -0.02  *(-0.37-0.34)* | -0.24  *(-0.55-0.13)* | 0.50^#^  *(0.17-0.72)* | 0.52^#^  *(0.20-0.74)* | -0.05  *(-0.40-0.31)* | 0.33  *(-0.03-0.62)* |
| *Right Medial,*  *BN 214* | -0.27  *(-0.57-0.10)* | -0.33  *(-0.61-0.03)* | -0.08  *(-0.42-0.29)* | 0.49^#^  *(0.17-0.72)* | 0.14  *(-0.23-0.47)* | -0.09  *(-0.43-0.28)* | -0.01  *(-0.37-0.35)* |
| **Dorsal Anterior Cingulate** |  |  |  |  |  |  |  |
| *Left Pregenual,*  *BN 179* | 0.43^#^  *(0.09-0.68)* | 0.42^#^  *(0.08-0.67)* | 0.40^#^  *(0.05-0.66)* | 0.62*  *(0.34-0.80)* | 0.30  *(-0.06-0.59)* | 0.40^#^  *(0.05-0.66)* | 0.11  *(-0.26-0.45)* |
| *Right Pregenual,*  *BN 180* | 0.54^#^  *(0.23-0.75)* | 0.40^#^  *(0.06-0.66)* | 0.62*  *(0.33-0.80)* | 0.60*  *(0.30-0.78)* | 0.20  *(-0.17-0.52)* | 0.34  *(-0.01-0.62)* | 0.16  *(-0.21-0.49)* |
| **Striatum** |  |  |  |  |  |  |  |
| *Left Dorsal*  *Caudate, BN 227* | 0.35  *(-0.01-0.62)* | 0.39  *(0.04-0.65)* | 0.36  *(0.00-0.63)* | 0.64*  *(0.36-0.81)* | 0.66*  *(0.40-0.82)* | 0.48^#^  *(0.15-0.71)* | 0.21  *(-0.16-0.52)* |
| *Right Dorsal*  *Caudate, BN 228* | 0.23  *(-0.14-0.54)* | 0.41^#^  *(0.06-0.67)* | 0.46^#^  *(0.13-0.70)* | 0.61*  *(0.32-0.79)* | 0.58^#^  *(0.29-0.78)* | 0.49^#^  *(0.16-0.72)* | 0.28  *(-0.08-0.58)* |
| *Left Ventral*  *Caudate, BN 219* | 0.30  *(-0.07-0.59)* | 0.44^#^  *(0.10-0.69)* | 0.47^#^  *(0.14-0.71)* | 0.63*  *(0.35-0.81)* | 0.44^#^  *(0.10-0.69)* | 0.56^#^  *(0.26-0.76)* | 0.24  *(-0.13-0.55)* |
| *Right Ventral*  *Caudate, BN 220* | 0.35  *(-0.01-0.63)* | 0.34  *(-0.02-0.62)* | 0.28  *(-0.08-0.58)* | 0.58^#^  *(0.28-0.77)* | 0.43^#^  *(0.08-0.68)* | 0.48^#^  *(0.15-0.71)* | 0.07  *(-0.30-0.41)* |
| *Left Nucleus*  *Accumbens, BN 223* | 0.03  *(-0.33-0.38)* | 0.06  *(-0.30-0.41)* | 0.07  *(-0.30-0.41)* | 0.76**  *(0.55-0.88)* | 0.35  *(-0.01-0.63)* | 0.64*  *(0.37-0.81)* | 0.26  *(-0.11-0.56)* |
| *Right Nucleus*  *Accumbens, BN 224* | -0.04  *(-0.39-0.32)* | -0.03  *(-0.39-0.33)* | 0.04  *(-0.32-0.39)* | 0.68*  *(0.43-0.83)* | 0.36  *(0.00-0.63)* | 0.45^#^  *(0.11-0.69)* | 0.27  *(-0.09-0.57)* |
| **Left Dorsolateral PFC** |  |  |  |  |  |  |  |
| *Left Dorsal Area*  *9/46, BN 15* | 0.41^#^  *(0.07-0.67)* | 0.48^#^  *(0.14-0.71)* | 0.66*  *(0.40-0.82)* | 0.68*  *(0.42-0.83)* | 0.50^#^  *(0.18-0.73)* | 0.35  *(-0.01-0.63)* | 0.21  *(-0.16-0.53)* |
| *Left Ventral Area*  *9/46, BN 21* | 0.35  *(-0.01-0.63)* | 0.47^#^  *(0.14-0.71)* | 0.41^#^  *(0.06-0.67)* | 0.61*  *(0.33-0.79)* | 0.24  *(-0.12-0.55)* | 0.03  *(-0.33-0.38)* | -0.03  *(-0.38-0.33)* |
| *Left Area 46,*  *BN 19* | 0.26  *(-0.11-0.56)* | 0.72*  *(0.50-0.86)* | 0.35  *(-0.01-0.63)* | 0.58^#^  *(0.28-0.78)* | 0.35  *(0.00-0.63)* | 0.04  *(-0.32-0.39)* | 0.33  *(-0.03-0.61)* |
| **Right Dorsolateral PFC** |  |  |  |  |  |  |  |
| *Right Dorsal Area*  *9/46, BN 16* | 0.06  *(-0.30-0.41)* | 0.19  *(-0.18-0.51)* | 0.11  *(-0.25-0.45)* | 0.54^#^  *(0.23-0.75)* | 0.15  *(-0.22-0.48)* | 0.31  *(-0.05-0.60)* | 0.20  *(-0.17-0.52)* |
| *Right Ventral Area*  *9/46, BN 22* | 0.37  *(0.02-0.64)* | 0.55^#^  *(0.24-0.75)* | 0.30  *(-0.06-0.60)* | 0.54^#^  *(0.23-0.75)* | 0.31  *(-0.06-0.60)* | 0.17  *(-0.20-0.50)* | 0.05  *(-0.31-0.40)* |
| *Right Area 46,*  *BN 20* | 0.21  *(-0.16-0.53)* | 0.46^#^  *(0.12-0.70)* | 0.14  *(-0.22-0.48)* | 0.55^#^  *(0.24-0.76)* | 0.36  *(0.01-0.63)* | 0.11  *(-0.26-0.45)* | 0.37  *(0.02-0.64)* |
| **Anterior Insula** |  |  |  |  |  |  |  |
| *Left Dorsal*  *Agranular, BN 167* | 0.44^#^  *(0.10-0.69)* | 0.39  *(0.04-0.65)* | 0.44^#^  *(0.10-0.69)* | 0.59^#^  *(0.30-0.78)* | 0.28  *(-0.08-0.58)* | 0.60*  *(0.31-0.79)* | 0.41^#^  *(0.06-0.67)* |
| *Right Dorsal*  *Agranular, BN 168* | 0.46^#^  *(0.13-0.70)* | 0.39  *(0.04-0.65)* | 0.51^#^  *(0.19-0.73)* | 0.50^#^  *(0.17-0.72)* | 0.17  *(-0.20-0.49)* | 0.52^#^  *(0.20-0.74)* | 0.01  *(-0.35-0.36)* |
| *Left Ventral*  *Agranular, BN 165* | -0.12  *(-0.46-0.24)* | -0.10  *(-0.44-0.26)* | -0.19  *(-0.51-0.17)* | 0.55^#^  *(0.24-0.76)* | -0.09  *(-0.43-0.27)* | -0.19  *(-0.51-0.18)* | 0.18  *(-0.18-0.51)* |
| *Right Ventral*  *Agranular, BN 166* | 0.11  *(-0.25-0.45)* | 0.35  *(0.00-0.63)* | 0.06  *(-0.30-0.41)* | 0.18  *(-0.18-0.51)* | 0.24  *(-0.13-0.55)* | 0.27  *(-0.09-0.57)* | 0.22  *(-0.14-0.54)* |

ICCs are consistent agreement and single-measure [i.e., ICC(3,1)]. ICCs between .4-.6 are denoted with a ^#^. ICCs between .6-.75 are denoted with a *. ICCs >.75 are denoted with a **. ICC value interpretation: poor (<.40), fair (.40-.59), good (.60-.74), excellent (≥.75). 95% confidence intervals are provided in parentheses below each ICC estimate. Negative ICCs are interpreted as having zero reliability. Abbreviations: ICC=intraclass correlation coefficient; ROI=region of interest; PFC=prefrontal cortex.

**Supplementary Table 4. Correlations of Test-retest Interval and Mean PSC Change / Adjusted ICCs for Composite ROIs**

|  | **Conflict Contrast (Decision-Making)** | | | **Negative Contrast (Outcome)** | | | **Reward Contrast (Reward)** | | |
| --- | --- | --- | --- | --- | --- | --- | --- | --- | --- |
| **Composite**  **ROI** | Pearson  Correlation | ICC(3, 1)  Original | ICC(3, 1)  Residuals | Pearson  Correlation | ICC(3, 1)  Original | ICC(3, 1)  Residuals | Pearson  Correlation | ICC(3, 1)  Original | ICC(3, 1)  Residuals |
| Amygdala | *r* = 0.20  (*p* = 0.29) | -0.15  *(-0.48-0.21)* | -0.14  *(-0.48-0.22)* | *r* = 0.09  (*p* = 0.63) | 0.60*  *(0.31-0.79)* | 0.60*  *(0.31-0.79)* | *r* = 0.13  (*p* = 0.51) | -0.03  *(-0.38-0.33)* | -0.04  *(-0.39-0.32)* |
| dACC | *r* = -0.25  (*p* = 0.19) | 0.51^#^  *(0.19-0.74)* | 0.54^#^  *(0.22-0.75)* | *r* = -0.50  (*p* < 0.005) | 0.63*  *(0.35-0.80)* | 0.70*  *(0.47-0.85)* | *r* = 0.03  (*p* = 0.89) | 0.41^#^  *(0.06-0.67)* | 0.41^#^  *(0.06-0.67)* |
| Striatum | *r* = 0.04  (*p* = 0.85) | 0.47^#^  *(0.14-0.71)* | 0.46^#^  *(0.13-0.70)* | *r* = -0.26  (*p* = 0.16) | 0.69*  *(0.45-0.84)* | 0.70*  *(0.46-0.85)* | *r* = 0.06  (*p* = 0.76) | 0.58^#^  (0.28-0.77) | 0.57^#^  (0.28-0.77) |
| Left  dlPFC | *r* = -0.22  (*p* = 0.25) | 0.48^#^  *(0.15-0.72)* | 0.50^#^  *(0.18-0.73)* | *r* = -0.54  (*p* < 0.005) | 0.66*  *(0.40-0.82)* | 0.74*  *(0.52-0.87)* | *r* = 0.13  (*p* = 0.50) | 0.14  *(-0.23-0.47)* | 0.14  *(-0.23-0.47)* |
| Right dlPFC | *r* = -0.08  (*p* = 0.66) | 0.28  *(-0.08-0.58)* | 0.29  *(-0.08-0.58)* | *r* = -0.67  (*p* < 0.005) | 0.49^#^  *(0.16-0.72)* | 0.67*  *(0.41-0.83)* | *r* = 0.24  (*p* = 0.20) | 0.27  *(-0.10-0.57)* | 0.27  *(-0.09-0.57)* |
| Anterior Insula | *r* = -0.10  (*p* = 0.61) | 0.11  *(-0.25-0.45)* | 0.07  *(-0.30-0.41)* | *r* = -0.33  (*p* = 0.07) | 0.54^#^  *(0.23-0.75)* | 0.58^#^  *(0.29-0.78)* | *r* = 0.06  (*p* = 0.77) | 0.31  *(-0.05-0.60)* | 0.29  *(-0.07-0.59)* |

Pearson’s correlations were conducted between each individual’s test-retest interval (i.e., days between time 1 and 2), and the subsequent residuals for the regression line were saved and used for ICC estimates. ICCs are consistent agreement and single-measure [i.e., ICC(3,1)]. ICCs between .4-.6 are denoted with a ^#^. ICCs between .6-.75 are denoted with a *. ICC value interpretation: poor (<.40), fair (.40-.59), good (.60-.74), excellent (≥.75). 95% confidence intervals are provided in parentheses below each ICC estimate. Negative ICCs are interpreted as having zero reliability. Abbreviations: PSC = percent signal change; ICC=intraclass correlation coefficient; ROI=region of interest; dACC=dorsal anterior cingulate cortex; dlPFC=dorsolateral prefrontal cortex.

**Supplemental Table 5. Correlations of Absolute Group Mean PSC at T1 and ICCs for ROI Subregions**

|  | Decision Making Trial Type | | | Outcome Trial Type | | Reward Trial Type | | All Trial Types |
| --- | --- | --- | --- | --- | --- | --- | --- | --- |
|  | *Approach* | *Avoid* | *Conflict-Averaged* | *Negative* | *Positive* | *Reward* | *No Reward* |  |
| Pearson Correlation | *r* = 0.37 | *r* = 0.38 | *r* = 0.45 | *r* = -0.52 | *r* = 0.36 | *r* = 0.29 | *r* = 0.02 | *r =* .17 |

Pearson’s correlations were conducted between absolute group mean PSC at T1 and the ICC estimates for the 22 composite ROI subregions for individual contrast for each phase of the task. Each individual trial type includes 22 data points while that contrast including all trial types has 154 data points. Abbreviations: PSC = percent signal change; T1 = time 1; ICC=intraclass correlation coefficient; ROI=region of interest.
